# Physiological fidelity of a satellite-derived forest resilience indicator in the Amazon

**DOI:** 10.1038/s41559-026-03116-z

**Published:** 2026-07-21

**Authors:** Kexin Song, James Knighton, Shi Qiu, Xiucheng Yang, Ji Won Suh, Julia V. Tavares, Yanlan Liu, Xiaonan Tai, Robert Fahey, Christopher S. R. Neigh, Russell Callahan, Falu Hong, Tian Li, Ashley Grinstead, Wei Ren, Chandi Witharana, S. Blair Hedges, Zhiqiang Yang, Rodrigo Vieira Leite, Paulo R. L. Bittencourt, Zhe Zhu

**Affiliations:** 1 Department of Natural Resources and the Environment, University of Connecticut, Storrs, CT USA; 2 Department of Geography, University of Victoria, Victoria, British Columbia Canada; 3 School of Geography, University of Leeds, Leeds, UK; 4 Department of Geography, University of California, Los Angeles, Los Angeles, CA USA; 5 Department of Biological Sciences, New Jersey Institute of Technology, Newark, NJ USA; 6 Biospheric Sciences Laboratory, NASA Goddard Space Flight Center, Greenbelt, MD USA; 7 Department of Earth Sciences, University of Connecticut, Storrs, CT USA; 8 Center for Biodiversity, Temple University, Philadelphia, PA USA; 9 US Forest Service, Rocky Mountain Research Station, Corvallis, OR USA; 10 School of Earth and Environment Sciences, Cardiff University, Cardiff, UK

**Keywords:** Ecophysiology, Forest ecology, Forestry, Environmental impact

## Abstract

Reliable resilience indicators are urgently needed to monitor global forest health under increasing climate stress. Lag-1 temporal autocorrelation (TAC) of satellite vegetation indices captures forest recovery speed and is widely used as a resilience metric, but its mechanistic underpinnings and methodological reliability have been questioned. Here we use Landsat observations and field data from the Amazon to investigate how satellite-derived TAC relates to in situ measurements of the hydraulic safety margin (HSM), a key physiological trait for plants’ capacity to accommodate drier conditions. We find a strong direct correlation between HSM and the resilience proxy (1-TAC) (*R*^2^ up to 0.57, *P* = 0.0116), providing evidence that forest recovery speed is constrained by drought resistance. A model incorporating both HSM and cumulative water deficit explains up to 83% of the variance in resilience, indicating that hydraulic strategies and local climate act as a joint, primary axis of forest resilience. Importantly, we show that this relationship is contingent on the selected vegetation index, the temporal frequency of satellite observations and the rolling window size. Taken together, our findings establish the physiological fidelity of TAC as a forest resilience metric and offer methodological guidance.

## Abstract (web summary)

This analysis shows that a widely used satellite-based ecosystem resilience indicator is a real signal linked to plant physiological responses to water deficit, provided that the estimation is based on appropriate methodological choice.

## Main

Forest ecosystems, a major terrestrial carbon sink on our planet, are under accelerating threats from a changing climate^1^ and human disturbances^2,3^. Rising temperatures^4,5^, intensifying droughts^6–9^, and land-use and land cover changes^10–12^ are pushing many forests towards their physiological limits, raising concerns about their capacity to recover from natural and anthropogenic stressors (herein resilience)^13^. As resilience declines, forests edge closer to tipping points where even small disturbances could trigger irreversible ecological collapse^14^, with far-reaching consequences for the global climate system and human well-being. Therefore, detecting early warning signals of resilience loss before such critical thresholds are crossed is one of the most urgent challenges of our time^15^.

**Fig. 1:**
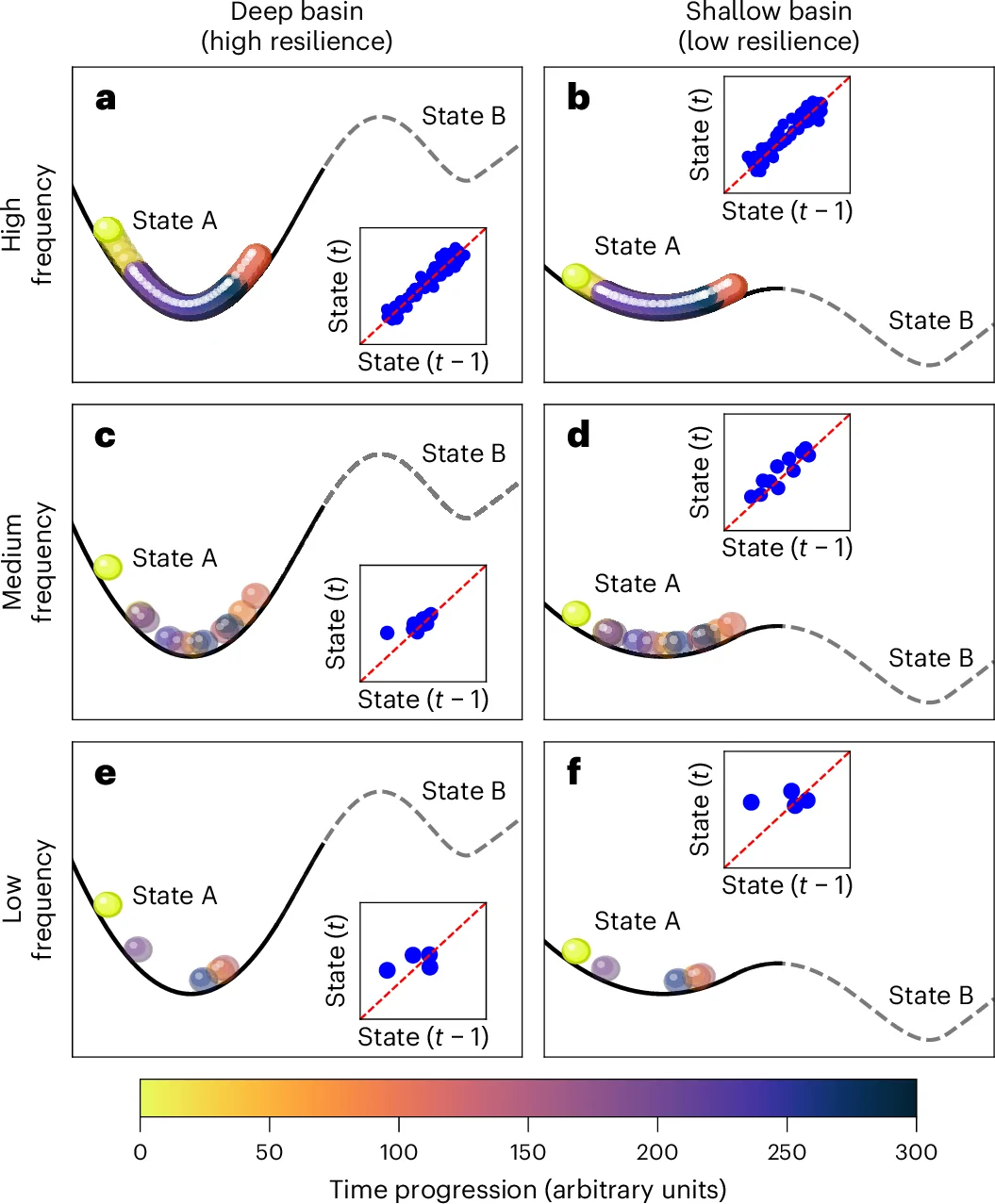
A conceptual basin stability diagram illustrates how observational frequency influences the detection of system dynamics (top, high frequency; middle, medium frequency; bottom, low frequency). **a**,**c**,**e**, The graphs represent a high-resilience system with a deep stability basin at high frequency (**a**), medium frequency (**c**) and low frequency (**e**). **b**,**d**,**f**, The graphs show a low-resilience system with a shallow basin at high frequency (**b**), medium frequency (**d**) and low frequency (**f**). The coloured spheres mark system states along stability basin A, with colour coding indicating the time of observation. Insets display lag-1 autocorrelation plots, that is, state at time *t* versus state at *t* − 1. Too much or not enough sampling of observations may misrepresent system intrinsic recovery rate when using lag-1 autocorrelation.

In recent years, satellite-based indicators have emerged as powerful tools to provide evidence of plant function and assess resilience at large spatial scales^16^. Among these, lag-1 temporal autocorrelation (TAC) has been widely adopted as a proxy for ecosystem resilience^4,5,8–10,17–22^. TAC quantifies the correlation between successive system states under naturally occurring stochastic fluctuations or ‘within-the-basin’ perturbations^23^ (Fig. 1, state A). An increase in TAC means the current state closely resembles the past, which indicates the system takes longer to recover from stressors and suggests that the system is approaching a tipping point^15^, where even small shocks could trigger abrupt catastrophic shifts^23,24^. This contrasts with traditional approaches that measure recovery rates and trajectories after major disturbances (events that push the ball ‘out of the basin’; Fig. 1, state B)^25–28^. Such events occur only in a small fraction of the land surface^29^ and typically assume an intact initial state^30^. While satellite TAC has been rapidly adopted to detect global resilience trends^5,17^, identify the drivers of resilience loss^4,31^ and forecast critical transitions and tipping points^8,32^, this uptake has outpaced the evaluation of its underlying physiological mechanisms. The link between TAC and resilience is supported by theoretical analyses, model simulations^33–35^, controlled experiments^15,36,37^ and biome-specific empirical studies^5,38–40^, and has been further investigated by comparing TAC to observed vegetation recovery following disturbances^41^. Yet, large-scale forest resilience assessments rely almost exclusively on TAC derived from satellite time series, often lacking the field-based physiological validation required to confirm these signals. This reliance on satellite time series is particularly concerning because, unlike controlled simulations, remote sensing is subject to multiple sources of uncertainty^16,18,42^. Atmospheric conditions, sensor-specific limitations and data-processing choices (for example, outlier removal and gap filling^42^), introduce noise that can alter TAC estimates^16,18,42^, complicate the ecological interpretation and drive the contrasting patterns of vegetation resilience trends reported in recent literature^30^. Consequently, a central question emerges: does satellite-observed TAC truly reflect a forest’s intrinsic recovery capacity, or does it primarily capture statistical artefact imposed by observation and processing constraints?

To understand the reliability of TAC, we must first place it within the formal framework of ecosystem stability^43^, which is a multifaceted concept^44^ encompassing multiple components, notably resistance—the capacity to withstand a stressor, and resilience—the ability and speed of recovery afterwards^45,46^. While resistance and resilience are sometimes used interchangeably in applied literature^47^, we adhere to their formal definitions. Unlike measures derived from large, state-altering disturbances, TAC is calculated from the minor fluctuations, or perturbations, that occur within an ecosystem’s stable state.

Plant resistance to drought can be inferred from hydraulic traits that set thresholds of hydration in which plants can operate without damage^48,49^. Embolism resistance, commonly represented as *P*_50_ (ref. ^50^) (the stem water potential at which 50% of stem water transport capacity is lost), captures a plant’s capacity to maintain the water transport system intact during water deficit periods. The hydraulic safety margin (HSM^51^) is defined as the distance from the plant water potential during the peak of the dry season (*P*_min_) to *P*_50_. Partial embolism of plant xylem may reflect a loss of some xylem vessels, which reduces conductance but not above the threshold that would result in mortality. Embolism that does not result in mortality cannot be reversed in the short term, and reduced hydraulic conductance would constrain forest recovery^52^. In this sense, plants operating at low HSM have increased exposure to xylem damage during drought and potentially need high recovery capability, whereas plants with high HSM maintain a larger safety buffer at the cost of maintaining a redundant safety margin during most periods^53,54^. Here, drought refers to seasonal decreases in water availability that are within the bounds of the local climate, rather than an exceptional event that potentially pushes an ecosystem beyond its tolerance. In addition, instead of treating HSM as a static species trait, we consider it a time-specific and context-dependent resistance metric of how closely plants function to their hydraulic threshold. As quantifying community-level HSM^49,55,56^ requires substantial field investment (Methods), such data have only recently become available, providing a rare opportunity to examine the mechanisms underlying satellite-derived TAC. Building on the hypothesis that resistance and resilience are closely linked, we propose that hydraulic traits conferring drought resistance are a fundamental precondition that enables and governs an ecosystem’s resilience^53,57,58^: forest plots with low HSM will show large temporal changes in their function indicated by high TAC, while plots with high HSM will show small temporal changes and low TAC. By explicitly linking TAC, a potential resilience metric, to HSM, we seek to provide the physiological explanation of resilience patterns at landscape scales.

**Extended Data Fig. 1:**
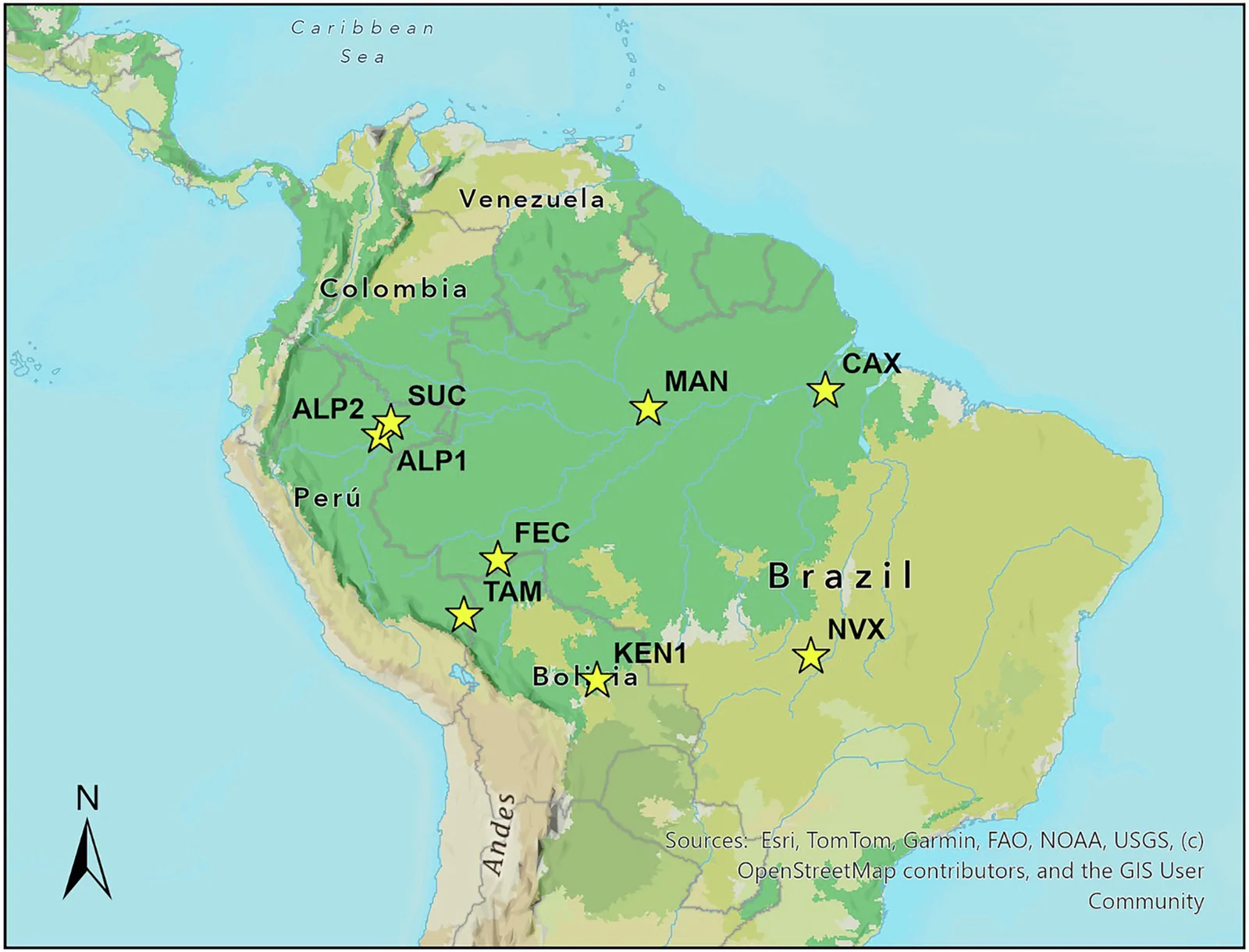
Geographic distribution of forest plots across South America where in-situ plant hydraulic trait measurements were collected by refs. ^49,55,56^. Plot codes (yellow stars) indicate individual plot names. For visualization purposes, the two geographically adjacent sites, ALP1 and ALP2, are shown under a common label (ALP). These sites represent a broad gradient of climatic and ecological conditions, providing ground-based data to evaluate remotely sensed ecosystem resilience. Their details can be found in Extended Data Table 1. Basemap from Esri (Esri, TomTom, Garmin, FAO, NOAA, USGS, © OpenStreetMap contributors, CNES/Airbus DS, InterMap, NASA/METI, NASA/NGS and the GIS User Community).

**Extended Data Fig. 2:**
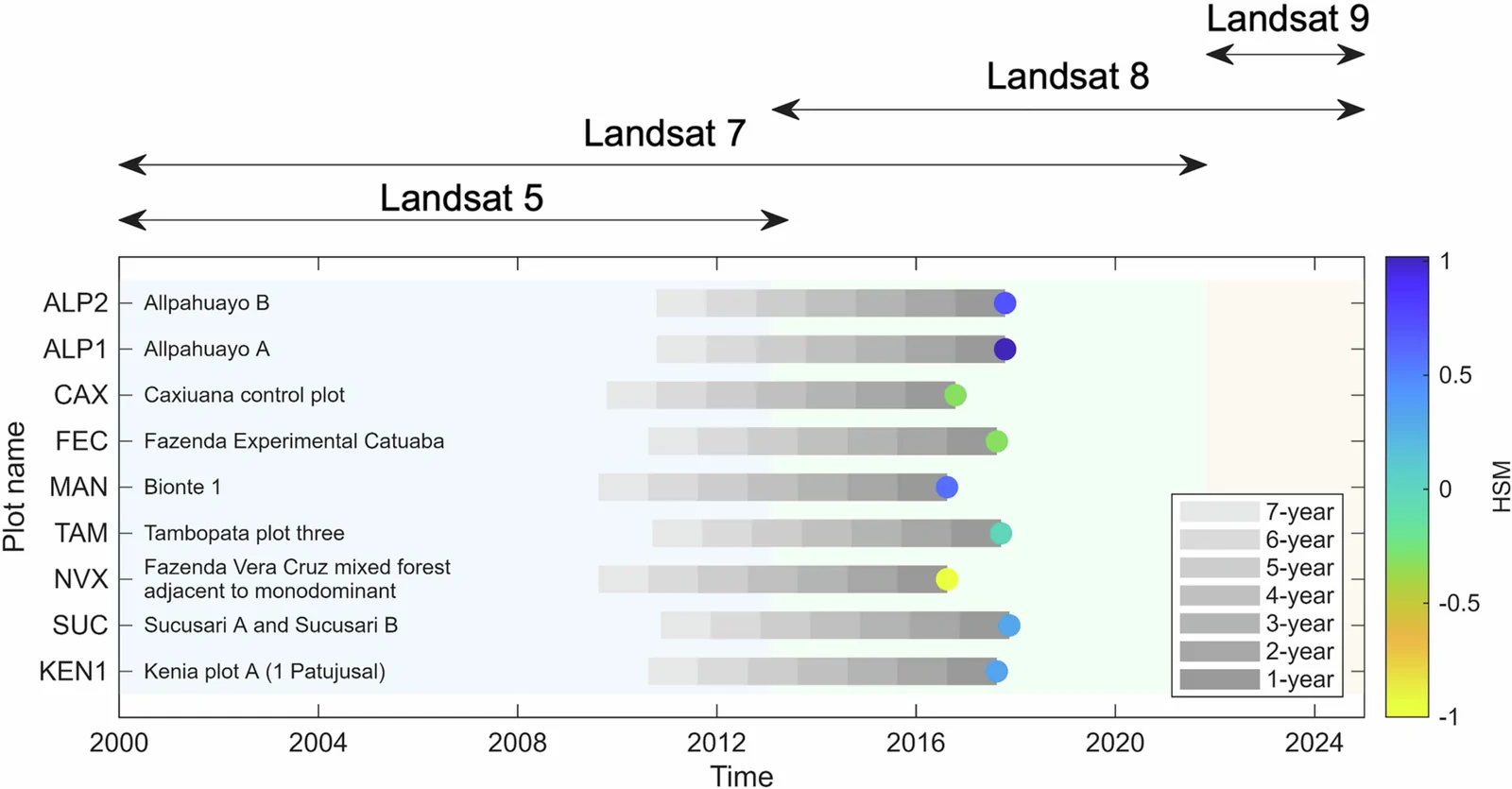
Field sample time and corresponding satellite temporal windows used for TAC calculation for each forest plot. Dots indicate the timing of field measurements of Pmin, with colors representing the community-level HSM values. Grey bars denote the rolling temporal windows of satellite observations used to compute TAC preceding each field sampling event, with darker shades indicating shorter windows and lighter shades representing longer windows. Colored background panels illustrate the periods of Landsat sensors included (blue: Landsat 5 and 7; green: Landsat 7 and 8; orange: Landsat 8 and 9), and arrows at the top indicate the operational timelines of these satellite sensors. HSM: hydraulic safety margin, defined as the difference between the minimum stem water potential (Pmin) and P50, the water potential at which 50% of xylem conductivity is lost.

The primary barrier to validating satellite-derived resilience indicators such as TAC has been a mismatch between the dynamic, landscape-scale nature of the satellite data and the static, species-level nature of available physiological data. This problem is compounded by a spatial mismatch: most previous resilience studies^4,5,19–21,40,59,60^ have relied on coarse-resolution sensors (for example, Moderate Resolution Imaging Spectroradiometer (MODIS) or Advanced Very High Resolution Radiometer (AVHRR)) whose large pixels average over vast, heterogeneous landscapes, making them spatially incompatible with plot-scale field measurements. While extensive HSM data exist in global compilations^58,61–63^, these datasets are not suitable for this purpose. They typically report species-mean traits as fixed constants, aggregated from disparate locations, years and measurement methods, and often without precise geolocations or sampling dates^64,65^. This creates spatiotemporal and methodological mismatches that make the data incompatible with the goal of validating a time-varying, community-specific metric such as TAC. To overcome this critical obstacle and bridge the gap between fine-scale physiological mechanisms and coarser-resolution global observations^66^, our study utilizes two key datasets: a collection of plot-specific, time-explicit HSM measurements from nine Amazonian forest plots (obtained from ref. ^49^, which also used data from refs. ^55,56^; see details in Extended Data Fig. 1 and Extended Data Table 1) and the 30-m spatial resolution of Landsat data. These datasets provide a rare opportunity to perform a direct, mechanistically grounded investigation by aligning co-located and co-timed satellite and field observations (Extended Data Fig. 2), enabling a robust test of the physiological basis of TAC as a forest resilience indicator. Without such investigation, we risk building a global understanding of ecosystem stability on a foundation of unverified approaches.

Here, we provide a direct, field-based investigation of the physiological basis underlying TAC as an indicator of forest resilience. We bridge the scale from global satellite pixels to individual plant function by comparing TAC with plant hydraulic traits from 114 species and more than 342 individual plants in Amazon forests across a precipitation gradient. Specifically, we test the hypothesis that forests composed of species with higher physiological resistance (that is, a wider HSM) suffer less hydraulic damage and functional loss during a drought. Consequently, they retain a greater capacity to recover quickly once conditions improve, thus exhibiting higher resilience (a lower TAC). Furthermore, by testing a suite of vegetation indices, temporal compositing schemes and rolling window lengths, we aim to identify the optimal methodological combination that consistently captures true resilience signals while avoiding systematic biases. This work seeks to establish a direct mechanistic link between a remotely sensed resilience proxy and field-based plant hydraulic functions, strengthening the ecological foundation for monitoring ecosystem resilience from Earth observations and providing an essential basis for developing robust early warning systems.

## Results

### Physiological evidence of TAC as an ecosystem resilience indicator

**Fig. 2:**
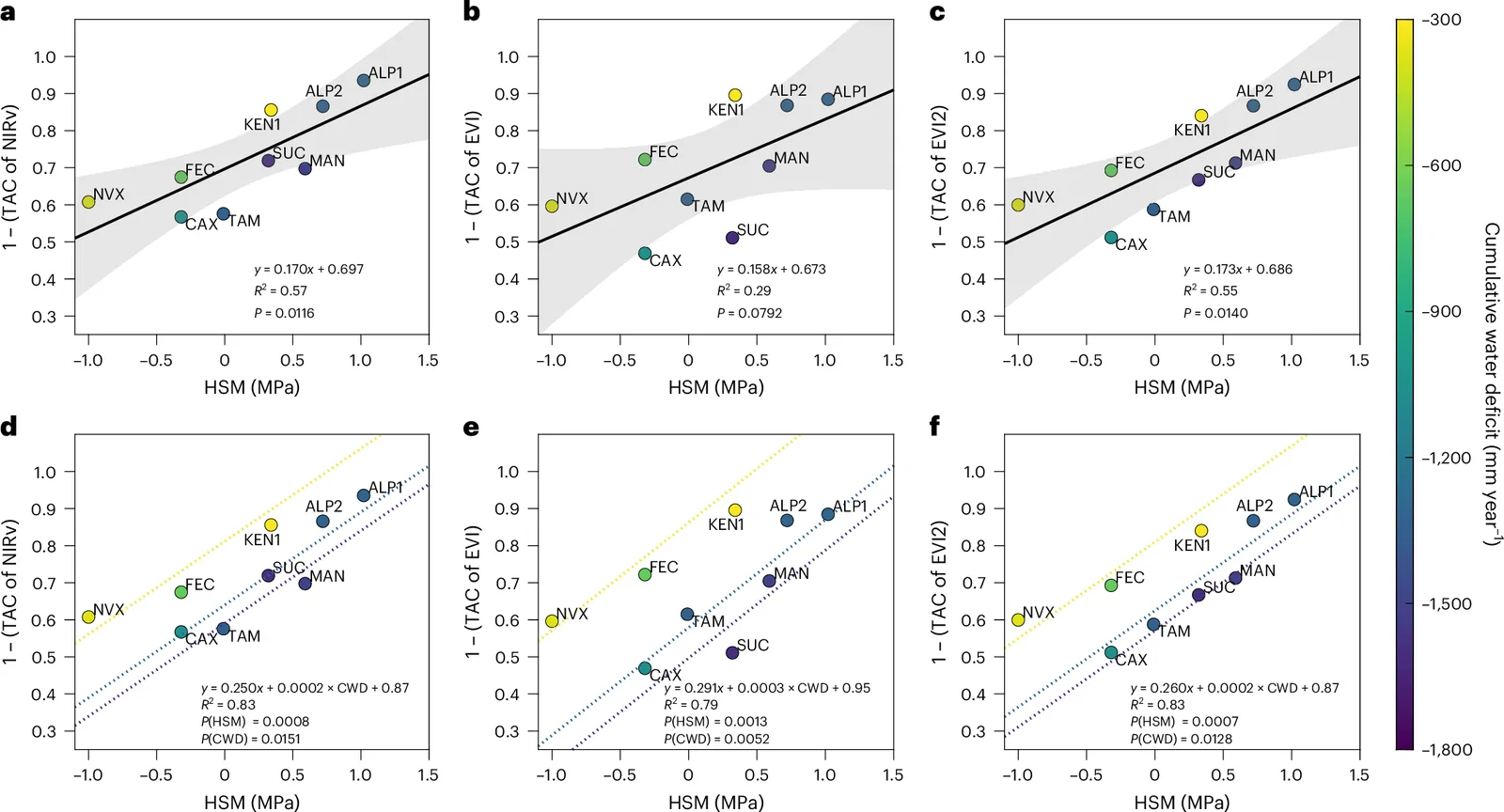
Relationships between a satellite-derived resilience proxy (1-TAC) and a field-measured resistance trait (HSM). Each point represents one of nine Amazonian forest sites, with resilience values paired to the basal-area weighted mean HSM at the plot level. The colour of the dot represents the cumulative water deficits (CWD; caculated as long-term average actual evapotranspiration minus precipitation, in mm year^−1^). **a**–**c**, The graphs show simple linear regressions where resilience is predicted from HSM alone, based on NIRv (**a**), EVI (**b**) and EVI2 (**c**). TACs were calculated using a bimonthly compositing interval and a 4-year rolling window. Shaded regions denote 95% confidence intervals for the fitted regressions. **d**–**f**, The graphs show multiple linear regressions in which CWD was added as a second predictor. The orange, green and blue lines represent the fitted models evaluated at maximum (**d**), median (**e**) and minimum CWD (**f**), respectively, illustrating how long-term water availability shifts the intercept of the resistance–resilience relationship. In the simple models, HSM explains 29–57% of the variance in resilience, while including CWD increases *R*^2^ to a range of 0.79–0.83. Across all models, HSM is positively correlated with 1-TAC, indicating that forests with greater resistance to hydraulic failure exhibit higher resilience. HSM is defined as the difference between the minimum stem water potential (*P*_min_) observed in the field at a specific year and *P*_50_, the water potential at which 50% of xylem conductivity is lost.

To evaluate the physiological basis of TAC as a generic indicator of ecosystem resilience, we compared 1-TAC (hereafter resilience proxies) derived from satellite vegetation indices against field-measured HSMs across nine forest plots^49,56,67^. We found that resilience proxies calculated from near-infrared reflectance of vegetation (NIRv), enhanced vegetation index (EVI), and two-band EVI (EVI2), all using a bimonthly temporal interval and a 4-year rolling window, were consistently and positively correlated with HSM (*R*^2^ from 0.29 to 0.57, *P* < 0.05 for NIRv and EVI2; Fig. 2a–c). These results provide direct evidence supporting our hypothesis that landscape-scale resilience captured by TAC is closely linked to the physiological drought resistance of the constituent trees quantified by HSM. Specifically, ecosystems exhibiting slower recovery from droughts tend to have a narrower HSM, reflecting a greater vulnerability to xylem damage and hydraulic failure.

As a tree’s HSM is an evolutionary adaptation shaped by its local climate, these resistance traits do not exist in a vacuum. Building on this physiological link, we next investigated how the relationship between resistance and resilience is jointly modulated by this broader environmental context. We incorporated cumulative water deficits (CWD) as an additional predictor in our models. The inclusion of CWD markedly improved the model fitness, with the full model explaining up to 83% of the variance in resilience^40,49,68^ (Fig. 2d–f). Both hydraulic resistance traits (*P* < 0.01) and long-term water deficit (*P* < 0.05) had significant effects. Sites under greater water deficit (warmer colours) tend to fall along parallel but upward-shifted regression lines, showing that at a given level of hydraulic resistance, drier forests exhibit systematically higher resilience. This pattern suggests that chronic water limitation favours plant communities with enhanced recovery potential, underscoring that resilience emerges not only from intrinsic hydraulic resistance traits but also from long-term adaptation to hydrological stress.

### Optimizing resilience monitoring with vegetation indices, temporal frequency and rolling window selections

**Fig. 3:**
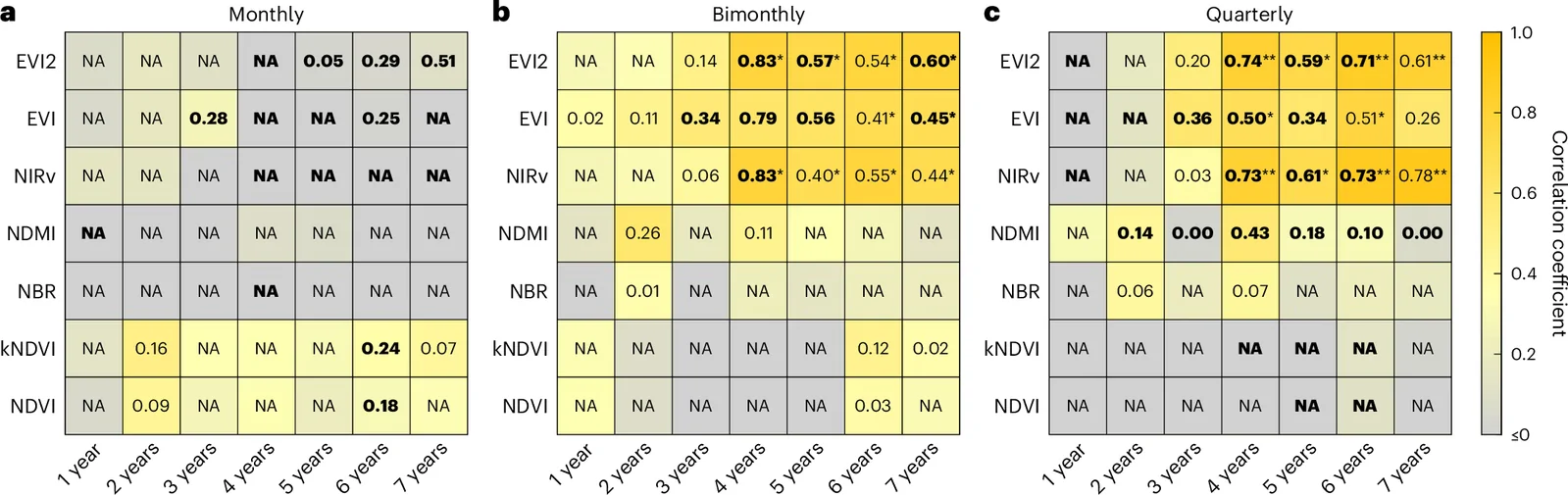
Methodological choices dictate the strength of the observed resistance–resilience relationship. **a**–**c**, Heat maps show the coefficient of determination (adjusted *R*^2^) for the relationship between a drought resistance metric (HSM) and a resilience proxy (1-TAC) across vegetation indices, rolling window lengths (1–7 years) and temporal compositing intervals: monthly (**a**), bimonthly (**b**) and quarterly (**c**). To ensure comparability across compositing schemes (constant sample size, *N* = 9), a consecutive gap-filling parameter (conseGap) of 1 was used for bimonthly and quarterly composites, and 2 for monthly composites. Cell colours represent Pearson correlation coefficients (*r*) from two-sided tests, with warmer tones indicating stronger positive correlations. Adjusted *R*^2^ values are shown only when both *r* and adjusted *R*^2^ are positive; otherwise, cells are labelled NA (not applicable). For each case, Akaike information criterion was used to determine whether to report the adjusted *R*^2^ from the simple regression (regular text) or from the multiple regression (bold), including CWD as an additional predictor. Asterisks denote significance (*P < 0.05 and **P < 0.01). Notably, the bimonthly interval indicates stronger and more consistent patterns. HSM is defined as the difference between the minimum stem water potential (*P*_min_) observed in the field at a specific year and *P*_50_, the water potential at which 50% of xylem conductivity is lost.

To optimize the TAC calculation and understand its sensitivity to methodological choices, we systematically tested the impacts of varying vegetation indices, temporal compositing intervals and rolling window sizes on the correlation with HSM. First, we found that the choice of vegetation indices influences the effectiveness of TAC as a proxy for drought resilience. NIRv, EVI and EVI2 yield more reliable outcomes with the bimonthly compositing scheme (Fig. 3b). This is because NIRv is more sensitive to vegetation structure and better captures the impact of environmental stress on plants^69^. EVI and EVI2 perform well in high biomass areas owing to their reduced saturation effects and are more resistant to atmospheric and soil background changes^70^. By contrast, the normalized difference vegetation index (NDVI), kernel NDVI (kNDVI), normalized burn ratio (NBR) and normalized difference moisture index (NDMI) showed weak or inconsistent correlations. NDVI saturates quickly in dense forests, limiting its ability to detect subtle stress signals. Although kNDVI was designed to mitigate this saturation issue, it still underperforms in dense vegetation compared with EVI. NBR, originally developed for burn severity detection, is more susceptible to acute canopy loss than to the gradual hydraulic decline relevant here. NDMI, which reflects canopy water content, is often affected by atmospheric noise and soil background, diluting its resilience signal. Further descriptions of each vegetation index can be found in Table 1.

**Table 1:** Description of vegetation indices used in this study

Indices	Full name	Equation	What it measures
NDVI^99^	Normalized difference vegetation index	$\mathrm{NDVI}\,=\,\frac{\mathrm{NIR}\,-\,\mathrm{RED}}{\mathrm{NIR}\,+\,\mathrm{RED}}$	Vegetation greenness and health; correlates with canopy density and photosynthetic activity^100,101^
kNDVI^102^	Kernel NDVI	${\mathrm{kNDVI}}\,=\,\tanh \,({{\mathrm{NDVI}}^{2}})$	Nonlinear variant of NDVI; reduces saturation and can be more robust to illumination/soil effects^102^
NIRv^103^	Near-infrared reflectance of vegetation	${\mathrm{NIRv}}\,=\,{({\mathrm{NDVI}}-0.08)}\,\times \,{\mathrm{NIR}}$	Proxy for vegetation NIR signal linked to gross primary production^104^. The 0.08 term is a soil adjustment to account for the NDVI of non-vegetated surface^103^.
NBR^105^	Normalized burn ratio	$\mathrm{NBR}\,=\,\frac{\mathrm{NIR}\,-\,\mathrm{SWIR}2}{\mathrm{NIR}\,+\,\mathrm{SWIR}2}$	Vegetation health/burn severity^106^
NDMI^107^	Normalized difference moisture index	$\mathrm{NDMI}\,=\,\frac{\mathrm{NIR}\,-\,\mathrm{SWIR}1}{\mathrm{NIR}\,+\,\mathrm{SWIR}1}$	Canopy/cell-water content^108^
EVI^109^	Enhanced vegetation index	${\mathrm{EVI}}\,=\,2.5\,\times \,\frac{{\mathrm{NIR}}\,-\,{\mathrm{RED}}}{{\mathrm{NIR}}\,+\,6\,\times \,{\mathrm{RED}}\,-\,7.5\,\,\times \,{\mathrm{BLUE}}\,+\,1}$	Vegetation greenness with improved sensitivity in high biomass and partial correction for aerosols/soil background^109^
EVI2^110^	Two-band enhanced vegetation index	${\mathrm{EVI}}2\,=\,2.5\,\times \,\frac{{\mathrm{NIR}}\,-\,{\mathrm{RED}}}{{\mathrm{NIR}}\,+\,{\mathrm{RED}}\,+\,1}$	A two-band version of EVI that has similar sensitivity in high biomass regions^110^

In terms of different temporal compositing intervals, we found that it must be appropriately matched to the ecosystem’s intrinsic recovery rhythms. A mismatch in either direction—sampling too frequently or too infrequently—obscures the true resilience signal. Unexpectedly, denser observation intervals (monthly; Fig. 3a) produced weak and non-significant correlations with field-based hydraulic data. This oversampling inflates TAC values by capturing high autocorrelation from short-term similarity (Fig. 1a,b) rather than the forest’s slower, intrinsic recovery rate^30^. This is akin to using a high-speed camera to film a slow-moving object; the near-identical consecutive frames create an artificially high autocorrelation, while masking the object’s true, long-term trajectory. This can misleadingly suggest a loss of resilience when the system is merely responding to minor, transient fluctuations. Conversely, when the observation frequency is too low (for example, quarterly; Fig. 3c), the satellite measurements become decoupled from the system’s actual dynamics. As shown in Fig. 1e,f, sparse observations serve as disconnected snapshots, failing to capture the coherent trajectory of perturbation and recovery. This is analogous to attempting to understand a bouncing ball by taking only two or three photos at random; the sparse data points cannot reveal the underlying physics of the rebound. Among all temporal settings, bimonthly compositing yielded the strongest and most consistent correlations (Fig. 3b). This frequency appears to achieve an optimal resonance, synchronizing the observation interval with the characteristic timescale of forest perturbation and recovery (shown in Fig. 1c,d). It is sufficiently long to suppress spurious autocorrelation introduced by oversampling, yet short enough to retain meaningful ecological dynamics and perturbation signals. Previous work^71^ also showed that 64-day TAC of evapotranspiration deficit was strongly related to evapotranspiration water age, a tracer of how long water resides within soil before being transpired, which highlighted that observation frequencies around 2 months could synchronize with the intrinsic memory of forest water fluxes. Therefore, selecting the correct temporal granularity is not a mere methodological detail but a fundamental prerequisite for translating satellite time series into a valid indicator of ecosystem resilience.

The choice of rolling window size further modulated these outcomes, revealing a critical trade-off between signal fidelity and temporal relevance. Shorter windows (1–3 years) were insufficient to distinguish the underlying ecological resilience signal from the short-term stochastic noise. Think of a short camera exposure in low light; these brief windows failed to integrate enough information to overcome the noise, resulting in weak and often non-significant correlations (Fig. 3). As the window length increased, the signal-to-noise ratio improved, allowing the true resilience signal to emerge. Statistically significant correlations peak at a 4-year window (Fig. 3b). This intermediate duration appears optimal, providing a long enough observational period to average out stochastic noise, and reveals the more persistent, mechanistically grounded resilience signal. However, extending the window beyond this optimal range (for example, 7+ years) yielded diminishing returns. Very long windows excessively smoothed the resilience signal—a phenomenon akin to an overexposed photograph losing the details of a specific moment—risking a decoupling of the TAC estimate from the contemporaneous resilience state captured by field measurements.

## Discussion

**Fig. 4:**
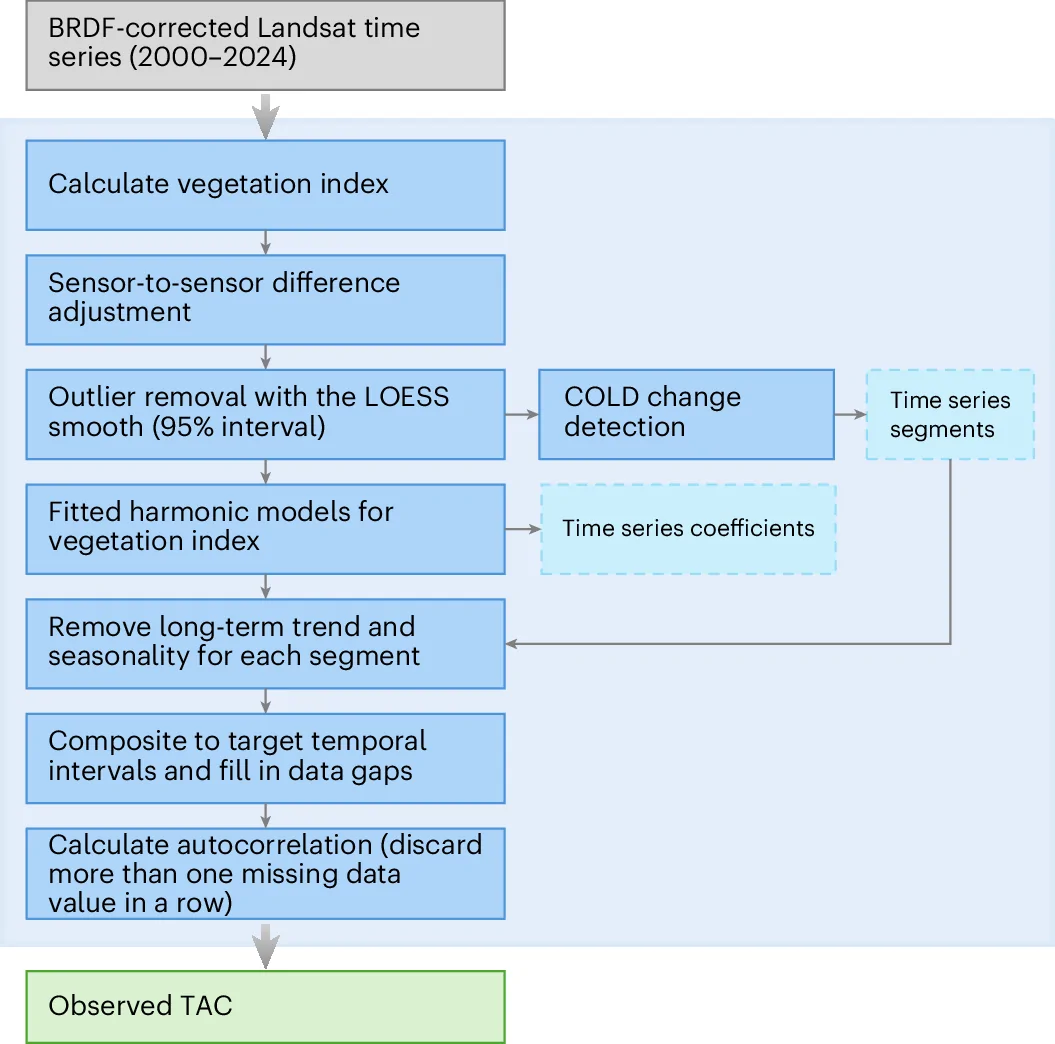
Workflow for deriving observed TAC from BRDF-corrected Landsat time series (2000–2024). The process includes vegetation index calculation, cross-sensor adjustment, outlier removal, harmonic modelling and time series segmentation using the COLD algorithm^96^, and detrending of long-term and seasonal components to obtain vegetation index residuals, followed by temporal compositing and gap-filling. Final TAC values are computed as lag-1 autocorrelation of the processed vegetation index residual time series. LOESS, locally estimated scatterplot smoothing; COLD: continuous monitoring of land disturbance.

This study provides a mechanistic explanation for a critical question in ecosystem science: whether satellite-derived TAC reflects ecologically meaningful resilience dynamics. Our results demonstrate that TAC is not merely a statistical value but also a landscape-scale expression of a forest’s stability strategy, which is determined by both intrinsic physiology and long-term climatic context. By linking a satellite-derived resilience indicator (TAC) with a field-measured resistance trait (HSM), we establish that the most resilient forests—those recovering fastest from non-catastrophic stressors—are those physiologically best equipped to resist stress^49,53^. Furthermore, we show that resilience emerges from a combination of this intrinsic resistance and long-term adaptation to local water availability^40,49,68^. The methodological workflow used to derive TAC from satellite observations is summarized in Fig. 4.

At the community level, one possible explanation for this observed mechanistic relationship between community-mean HSM and TAC is that each plot is occupied by a range of tree clades and tree ages representing a range of HSMs. With decreasing mean HSM, it is possible that there is an increasing fraction of trees that experienced irreversible damage or a loss of canopy^72,73^, contributing to slower post-drought recovery overall. Alternately, both resistance and resilience to drought may be jointly influenced by variations in water acquisition strategies. For example, deeply rooted trees or trees in downslope riparian areas that rely on groundwater rather than shallow soil moisture may maintain higher HSM during drought and therefore also recover more rapidly^74^. By establishing this link at the 30-m scale of Landsat observations, these findings strengthen TAC’s utility as a valid indicator for monitoring the health of global forests.

Our findings also clarify the specific ecological processes that satellite-derived TAC can monitor. The TAC metric, calculated from residuals around a stable state, primarily reflects the speed of physiological recovery within a surviving forest canopy. It quantifies how rapidly plants resume functioning after a non-lethal stress event. Our results show that this recovery speed is constrained by the plants’ capability to resist hydraulic damage in the first place. By contrast, a long-term increase in TAC at a given location would indicate a loss of resilience, probably reflecting a gradual structural shift driven by accumulating damage, such as irreversible xylem cavitation or low-level mortality, and slow replacement. Crucially, this interpretation also defines the boundaries of what TAC can measure. TAC is an early warning indicator of declining stability within a forest state that may signal elevated mortality risk. It is not a measure of recovery following a catastrophic, stand-replacing disturbance that would shift the ecosystem to an entirely new state (for example, grassland or an early successional forest).

**Extended Data Fig. 3:**
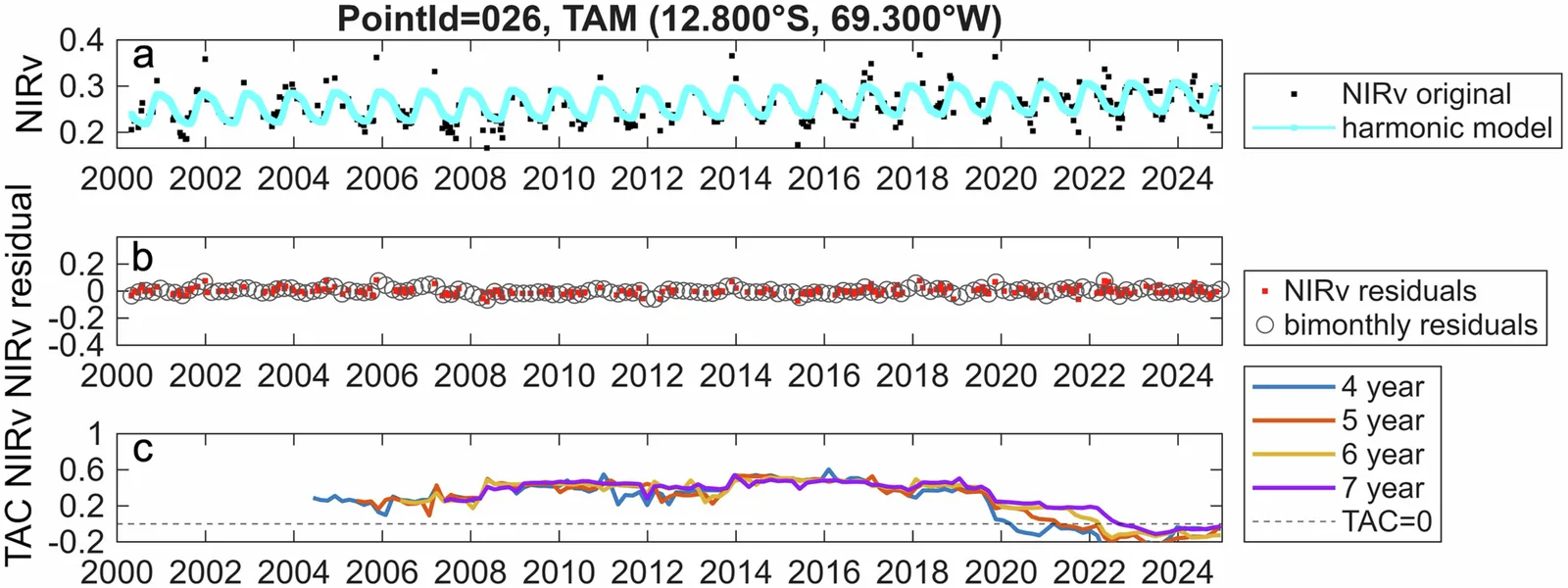
Time series analysis of near-infrared reflectance of vegetation (NIRv) for Tambopata plot three (TAM) (12.800°S, 69.300°W). **a**: NIRv observations (black points) and estimated harmonic model (cyan line). **b**: NIRv residuals (red points) and resampled bimonthly residuals (gray circles). **c**: Temporal autocorrelation (TAC) of bimonthly NIRv residuals at different rolling window lengths (colors correspond to 4–7 years). The dashed line represents TAC = 0.

To test the operational bounds of this indicator before such a catastrophic shift, we tracked its continuous trajectory across a severe decennial drought at Tambopata plot three (Extended Data Fig. 3). Crucially, this satellite time series is anchored by field data: during the drought peak in September 2017, the forest reached a critical physiological limit (HSM of −0.01 MPa, indicating a 50% loss of xylem conductance). As the TAC signal successfully tracked the ecosystem right down to the edge of hydraulic failure without decoupling into noise, we demonstrate that the indicator maintains its functionality even at the extreme bounds of physiological tolerance. However, because field-measured hydraulic traits require labour-intensive campaigns, they are currently limited to static snapshots (Extended Data Fig. 2), necessitating our use of a spatial gradient to establish this baseline. Fully bounding TAC across different climatic transitions will ultimately require pairing time series of satellite observations with continuous, in situ physiological monitoring. Evaluating dynamic, temporal changes in TAC as forests transition from well-hydrated states into extreme drought remains an important next step.

**Fig. 5:**
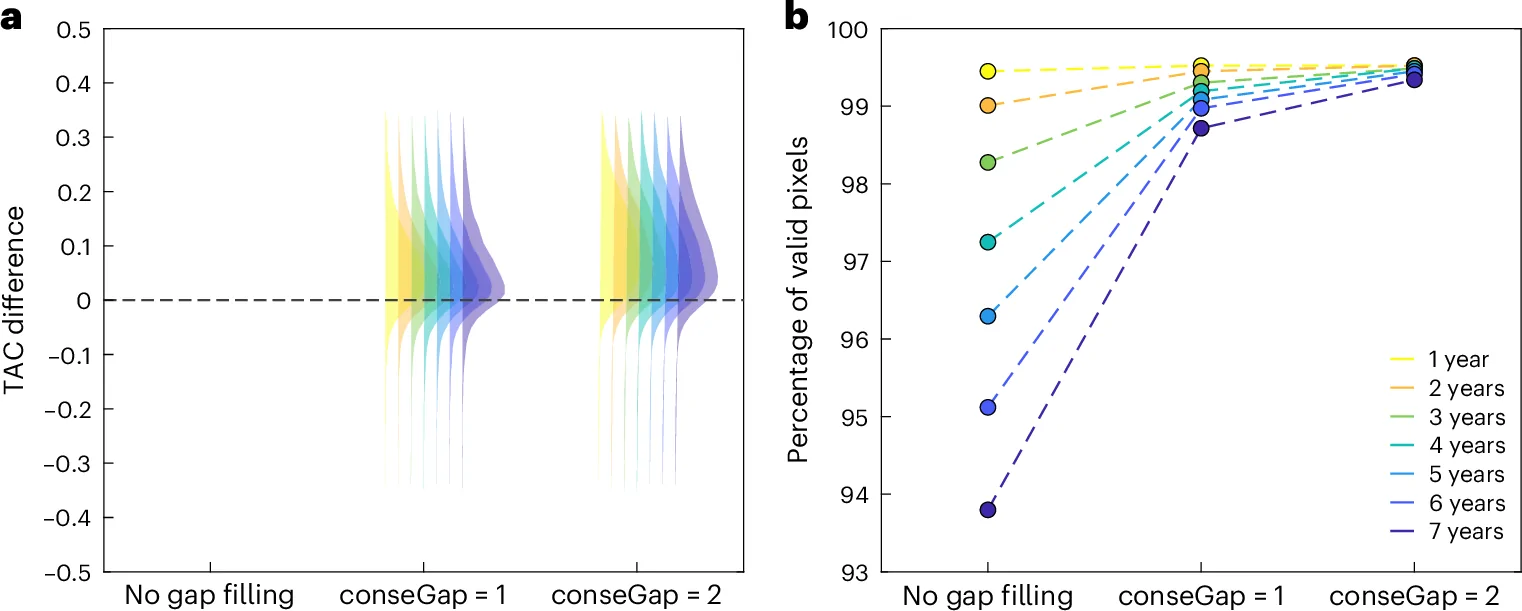
Sensitivity of TAC estimates to consecutive gap-filling thresholds at the bimonthly interval and across rolling windows. **a**, The distribution of long-term TAC differences between gap-filled scenarios (conseGap = 1 and conseGap = 2) and the no-gap-filling baseline across 1–7-year rolling windows. The half-violin plots show the density of pixel-level TAC differences, with colours indicating rolling-window length. The dashed horizontal line marks zero. **b**, The percentage of valid pixels retained under each gap-filling threshold relative to the total pixel count. The points and dashed lines connect results across rolling windows (1–7 years). Allowing up to one or two consecutive missing observations increases data availability while introducing limited artefacts to long-term TAC estimates.

**Extended Data Fig. 4:**
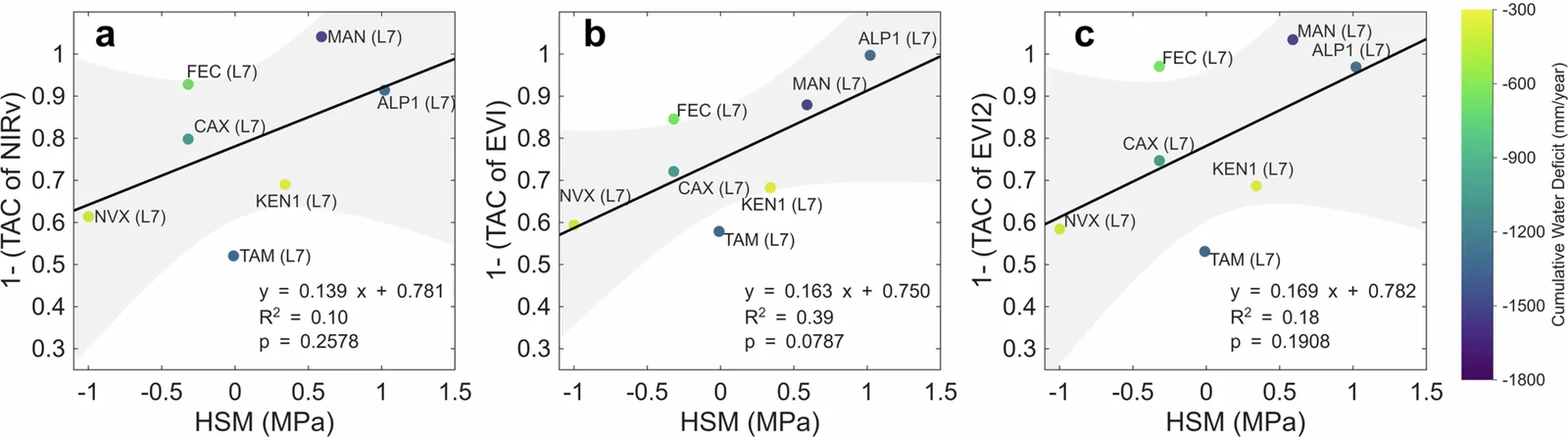
Relationships between a Landsat 7 (L7)-derived resilience proxy (1-TAC) and a field-measured resistance trait (HSM). Dot color represents the cumulative water deficit (CWD; the long-term average actual evapotranspiration - precipitation, mm/year). Panels (**a**–**c**) show simple linear regressions where resilience is predicted from HSM, based on **a**. NIRv, **b**. EVI, and **c**. EVI2. TACs were calculated using a bi-monthly compositing interval and a 4-year rolling window. Shaded regions denote 95% confidence intervals for the fitted regressions. TAC: lag-1 temporal autocorrelation. HSM: hydraulic safety margin, defined as the difference between the minimum stem water potential (Pmin) observed in the field at a specific year and P50, the water potential at which 50% of xylem conductivity is lost. NIRv: near-infrared reflectance of vegetation. EVI: enhanced vegetation index. EVI2: two-band EVI.

Beyond the biological insights, our work reveals that the reliability of satellite-derived ecological indicators is critically dependent on the careful synchronization of observation strategy with ecosystem-specific functional rhythms. The choice of vegetation indices, temporal compositing interval, rolling window of Earth observations and gap-filling strategies (Figs. 3 and 5) can drastically influence the reliability of TAC. We found that a bimonthly compositing interval achieved an optimal resonance, synchronizing the observational frequency with the characteristic timescale of forest perturbation and recovery. However, because functional rhythms and physical structures differ across biomes, these parameter settings cannot be universally applied. For instance, optimal temporal settings must adapt to inherent system dynamics^30^, as grasslands exhibit faster growth and shorter turnover rates than forest. Similarly, the choice of vegetation index must account for physical constraints, as high-biomass regions (for example, dense tropical forests) present well-known saturation challenges for accurate TAC-based resilience estimation^75–77^. Furthermore, because resilience metrics are highly sensitive to instrumental noise, combining data from multiple satellite missions requires rigorous cross-sensor harmonization. Our sensitivity analysis confirms that appropriately harmonized multisensor time series yield TAC estimates highly consistent with single-sensor baselines, successfully preserving the ecological signal while maximizing necessary temporal data density (Extended Data Fig. 4). This methodological finding provides a prerequisite for translating satellite time series into a valid indicator of ecosystem resilience and mandates a critical re-evaluation of trends reported in earlier studies that used different methodological combinations.

While this study provides the field-based evidence for the physiological basis of TAC, it is crucial to situate these findings within a broader context and define the path forward. We acknowledge that the observed correlations, though statistically significant, do not imply direct causality and that TAC, similar to any single metric, is a simplified representation of complex ecosystem dynamics. Our models establish hydraulic traits and background water availability as a primary axis of forest resilience, explaining up to 83% of the variance. However, because TAC ultimately captures all fluctuations in canopy greenness, the remaining unexplained variance probably reflects concurrent factors such as light competition, microclimate variations and pest outbreaks. Tracking resilience globally is critical; however, validating highly sensitive satellite metrics requires strict spatiotemporal data. Current global hydraulic syntheses^58,62,78^ often lack exact sampling timestamps, aggregate distinct plots and mix fundamentally different *P*_50_ measurement techniques. Therefore, we restricted our study to the Amazon—a uniquely standardized dataset^49^ serving as a highly controlled natural experiment. Although geographically constrained, the biophysical mechanisms linking xylem embolism to canopy degradation are governed by universal physiological principles, making our validation of TAC’s mechanistic fidelity generalizable.

This study also highlights the urgency to link species- or community-level hydraulic measurements with long-term Earth observations to understand ecosystem health and resilience. Species-specific traits, such as xylem vulnerability, turgor loss point, root depth and water-use efficiency, provide mechanistic insights into the adaptability of individual plants or communities, but are often limited in spatiotemporal extent and scalability. By contrast, satellite data enable seamless, continuous assessment of vegetation response to environmental stress but at a cost of low mechanistic understanding. Consequently, blindly applying satellite resilience products without verifying their underlying biological mechanisms risks misinterpreting how ecosystems actually respond to climate stress. Bridging the gap between remotely sensed monitoring and the physiology of actual trees in the field remains a key challenge for the progress of the discipline^66^. As demonstrated by our framework, achieving this integration requires a massive expansion of synchronized field campaigns. Future research must prioritize expanding continuous, standardized in situ monitoring and leveraging existing field datasets, such as forest inventories, flux towers and networks of global vegetation physiological traits (for example, the Xylem Functional Traits Database^61^ and TRY^79^), sap flux measurements (for example, Sapfluxnet^80^) and water potential (for example, PSInet^81^). Validation against ground-based measurements is crucial for ensuring that satellite-derived metrics reliably reflect the true ecosystem status with respect to changing climate and shifting disturbance regimes.

## Methods

### Hydraulic traits compilation

To assemble a robust dataset of plant hydraulic traits, we conducted an extensive literature review of peer-reviewed studies published since 2000. Our focus was on three key parameters widely used to assess in situ plant vulnerability and resistance to water stress: (1) the xylem water potential at 50% loss of hydraulic conductivity (${P}_{50}$), a direct measure of xylem vulnerability to embolism, (2) midday leaf water potential during the dry season (${P}_{\min }$), which reflects actual plant water stress under field conditions and is time specific, and (3) the HSM, defined as the difference between ${P}_{\min }$ and ${P}_{50}$. Among these, we prioritized HSM as a key physiological trait related to drought resistance, providing a powerful basis for investigating the mechanistic underpinnings of satellite-derived resilience metrics, because it quantifies how close a plant operates to its hydraulic failure threshold, which leads to drought-induced mortality^82,83^. Our final dataset^49,55,56^ comprises 114 tree species from nine sites within the Amazon basin, a region of global ecological importance known for its vulnerability to drought. These sites were specifically selected to represent a broad gradient of climatic and ecological conditions, from ever-wet to seasonally dry forests, with mean annual precipitation ranging from 1,418 mm to 2,087 mm and mean annual temperature from 23.46 °C to 26.63 °C, providing a powerful natural experiment for testing the TAC indicator.

We searched databases, including Google Scholar, Web of Science and Scopus, using keywords such as ‘hydraulic safety margin’, ‘p50’, ‘xylem vulnerability’ and ‘water potential’. While many studies reported hydraulic traits at the species level, only a few met the criteria necessary for direct comparison with satellite observations under spatial and temporal alignment. Studies were included if they (1) reported the sampling location and year, (2) described the same methods for measuring hydraulic traits and (3) included data collected from natural field conditions at a spatial scale covering at least one Landsat pixel (30 m by 30 m). Data were extracted directly from tables and supplementary materials. The spatial distribution of the selected plots is shown in Extended Data Fig. 1, and the compiled hydraulic dataset and its metadata can be found in the Extended Data Table 1.

### Preprocessing of Landsat time series

For this study, we exclusively used the Landsat time series. This choice was critical for three reasons. First, Landsat’s 30-m spatial resolution enables a direct spatial correspondence between the satellite pixels and the ecological scale of our field plots (0.44–1 ha). This alignment is impossible with the coarse-resolution (250 m to 1 km) sensors typically used in large-scale resilience studies. Second, this fine spatial resolution is essential for capturing the known heterogeneity of drought impacts on the Amazon. Coarse pixels would average the response of stressed and unstressed vegetation, fatally obscuring the specific, plot-level signal required to make a meaningful comparison with our in situ HSM measurements. Third, the consistent near-nadir viewing geometry of Landsat minimizes the radiometric noise from the bidirectional reflectance distribution function (BRDF) effects that are prominent in wide-swath, coarse-resolution sensors^84^. This ensures a higher signal-to-noise ratio, which is a fundamental prerequisite for detecting the subtle, residual fluctuations used in TAC calculation.

We extracted Landsat surface reflectance time series from Google Earth Engine to maintain reproducibility and cloud-based processing scalability. To ensure a consistent time series suitable for TAC analysis, we implemented a multistep preprocessing pipeline involving nadir BRDF adjustment, vegetation indices calculation, sensor-to-sensor harmonization and outlier removal based on locally estimated scatterplot smoothing (LOESS)^85^. The preprocessing pipeline transformed raw Landsat observations from Google Earth Engine into a harmonized, ecologically meaningful time series dataset.

We began by applying the *c*-factor BRDF normalization^86,87^ with optimized solar angles^88^ to raw surface reflectance data to account for differences in solar and viewing geometry. This correction ensured that reflectance values are comparable across dates and seasons^89^ and reduced the minor effects of Landsat 7 orbital drift^90^, providing a stable baseline for downstream analysis. Then, we calculated the NDVI, kNDVI, NBR, NDMI, NIRv, EVI and EVI2 time series using multispectral bands from Landsat 5–9 (Table 1). The vegetation index time series formed the basis for TAC metrics, capturing vegetation dynamics at a resolution of 30 m. To address sensor-to-sensor differences, we performed robust linear regression on observation pairs collected from 10,000 randomly sampled pixels within the geographic bounding box within 24° S to 12° N and 34° W to 82° W. The observation pairs are defined as Landsat 7 Enhanced Thematic Mapper Plus and Landsat 8/9 Operational Land Imager data acquired on the same day, given the similarity between Landsat 5 Thematic Mapper and Landsat 7 Enhanced Thematic Mapper Plus^91^. This step normalized Landsat 5/7 reflectance and vegetation indices to Landsat 8/9-like values, ensuring that differences introduced by sensor transitions did not bias TAC values. Finally, to improve signal fidelity, we removed extreme outliers on the basis of LOESS-smoothed confidence intervals (95%) in the kNDVI series. We chose kNDVI for this process because of two reasons: (1) picking a single vegetation index for outlier removal guarantees the same number of observations are retained across different vegetation indices and (2) kNDVI is more resistant to illumination and soil effects, making it more robust in heterogeneous forest environments. These refinements reduced noise in the time series and minimized TAC fluctuations caused by low-quality data. For temporal consistency, we also excluded Landsat 7 observation collected after the launch of Landsat 9.

### Climate data and forest cover

Climate variables used in this study were obtained from the ERA5-Land reanalysis product provided by the Copernicus Climate Change Service^92^. ERA5-Land offers monthly climate data at a spatial resolution of 0.1° (~9 km). We extracted four key variables relevant to ecosystem function and vegetation dynamics: 2-m air temperature, total precipitation, downwards solar radiation and water deficit (calculated as total ET minus total precipitation). Each climate variable was characterized using three statistical descriptors—mean, variance and lag-1 autocorrelation—capturing long-term climate background, interannual variability and short-term memory, respectively. Across forest plots, climate conditions spanned a broad gradient, with mean annual precipitation ranging from 1,418 mm to 2,087 mm, mean annual temperature from 23.46 °C to 26.63 °C and CWD from −1,688 mm to −316 mm year^−1^. Here, CWD represents the long-term average annual evapotranspiration minus precipitation over 2000–2024. Unlike the maximum climatological water deficit^93^ often used to isolate seasonal stress, our CWD captures the background water budget to represent the baseline hydrological conditions the forest is adapted to.

Forest cover data were obtained from the Global 2010 Tree Cover dataset^94^, selected for its high spatial resolution (30 m). Only pixels with more than 10% forest cover were defined as valid forest sample for subsequent analysis. These data are also used in the random forest (RF) model to factor out climate autocorrelation’s impact in the observed TAC time series (‘Enhanced TAC of vegetation indices’).

### Observed TAC of vegetation indices

We calculated the TAC at the locations of hydraulic trait measurements using 2000–2024 Landsat time series. We tested seven commonly adopted vegetation indices representing canopy greenness and health, including NDVI, kNDVI, NIRv, NBR, NDMI, EVI and EVI2. The overall workflow can be found in Fig. 4, and a time series example illustrating this calculation process can be found in Extended Data Fig. 3. Before TAC estimation, we removed long-term trends and seasonality from each vegetation index. This was carried out by fitting three-order harmonic models for each spectral band and vegetation index band using the Continuous Monitoring of Land Disturbance (COLD) algorithm^95,96^. This algorithm was chosen for its proven ability to robustly model phenological cycles and identify non-phenological breaks (disturbances) in noisy time series, thereby ensuring that TAC calculations were performed on residuals representing true ecosystem anomalies. For each observation date, COLD generates spectral estimates on the basis of the historical time series, as shown in equation (1), and flags a ‘temporal break’ when six consecutive observations deviate from model predictions beyond a predefined threshold (we used the default value of 0.99). This process serves two purposes: (1) disturbed pixels are excluded from further analysis, and (2) the full time series is segmented to allow accurate removal of linear trends and seasonal signals. Once the harmonic model was built, the model coefficients were used to filter out linear trend component and seasonal variations, including unimodal, bimodal and trimodal cycles. The resulting residual vegetation index time series were subsequently used for TAC calculation. Note that temporal breaks were detected using the Landsat surface reflectance bands; these break dates were then used to segment the time series of all derived vegetation indices before fitting their respective harmonic models.

1 $${\hat{\rho}}_{x}\,=\,{a}_{0}\,+\,\mathop{\sum}\limits_{k\,=\,1}^{3}\left\{{a}_{k}\cos \left(\frac{2\pi }{T}\right)\,x\,+\,{b}_{k}\cos \left(\frac{2\pi }{T}\right)\,x\right\}\,+\,{c}_{1}x,$$

where ${\hat{\rho }}_{x}$ is the modelled surface reflectance or vegetation index, x is Julian date, *k* is the temporal frequency of seasonality component (*k* = 1, 2 and 3), *T* is the number of days per year, *a*_0,*i*_ is the overall value of the *i*th band, ${a}_{k}$ and ${b}_{k}$ are the coefficients of intra-annual variations and ${c}_{1}$ is the interannual trend.

Moreover, we resampled vegetation index residuals into monthly, bimonthly and quarterly composites to generate evenly spaced time series. Different compositing intervals capture ecosystem fluctuations at varying temporal frequencies. Missing values were gap-filled using linear interpolation between adjacent time steps. To minimize artefacts from excessive interpolation, we excluded data pairs with more than one consecutive missing value. This threshold was selected to retain a sufficient number of valid pixels while preserving the accuracy of TAC estimation (Fig. 5). In addition, to assess temporal scale effects, we estimated TAC using rolling windows of 1–7 years. In this approach, TAC is calculated within a fixed-length window, and then the window is shifted forward step by step across the series, generating a continuous record of TAC dynamics. This allowed us to identify the temporal scale at which TAC best relates to forest resistance to drought (Extended Data Fig. 2).

### Enhanced TAC of vegetation indices

To isolate the vegetation’s intrinsic resilience from autocorrelation driven by climate variables, we applied the ‘space for time’ assumption described in ref. ^5^. This method reduces the confounding effect of climate autocorrelation on the observed TAC of vegetation indices, yielding an enhanced measure of ecosystem recovery rate. Specifically, we built random forest (RF) models with 100 trees that learned to predict observed TAC from a suite of climate predictors, forest cover and phenological dynamics indicated by COLD model coefficients; by holding the climate autocorrelation term constant at a baseline value (year 2000) and predicting again, we can estimate and subtract the portion of TAC attributable solely to climate effects. In this framework, the observed TAC (${\mathrm{TAC}}_{\mathrm{obs}}^{t}$) can be expressed as the sum of two components (equation (2)):

2 $${\mathrm{TAC}}_{\mathrm{obs}}^{t}\,=\,{\mathrm{TAC}}^{t}\,+\left.{\mathrm{TAC}}^{t}\,\right|\,{X}_{\mathrm{ac}}.$$

Here, ${\mathrm{TAC}}^{t}$ represents the intrinsic (ecosystem-driven) component, $\left.{\mathrm{TAC}}^{t\,}\right|\,{X}_{{ac}}$ reflects the contribution from autocorrelated climate variables ${X}_{\mathrm{ac}}$. To estimate these components, we trained RF regression models using ${\mathrm{TAC}}_{\mathrm{obs}}^{t}$ as response variables. Training samples were selected from forest pixels (>10% cover) within 500 km of each validation plot, drawn from an initial pool of 10,000 randomly sampled pixels. This ensured the training data reflected local climate and vegetation conditions. Predictor variables included climate factors (2-m air temperature, total precipitation, water deficit and downwards solar radiation), forest cover and COLD model coefficients (${a}_{0},\,{a}_{1},\,{a}_{2},\,{a}_{3},\,{b}_{1},\,{b}_{2},\,{b}_{3}\,\mathrm{and}$ ${c}_{1}$ in equation (1)), which capture the complete baseline phenology and long-term trajectory of each pixel. For each climate variable, we derived three terms, including mean, variance and lag-1 autocorrelation, to characterize background climate conditions, climate variability and climate autocorrelation, respectively. Thus, a total of 21 input variables were included. After training, we fixed the climate autocorrelation term to its baseline value (year 2000) and recalculated model predictions. The estimated climate autocorrelation effect can be derived by the difference between the original RF predictions and the recalculated ones (equation (3)). Last, we subtracted this effect term from the observed TAC, yielding an enhanced TAC metric that isolates the vegetation’s intrinsic resilience (equation (4)).

3 $$\left.{\mathrm{TAC}}^{t}\,\right|\,{X}_{\mathrm{ac}}\,=\,\mathrm{RF}\,\left({X}^{t}\right)\,-\,\mathrm{RF}\,\left({X}_{-\mathrm{ac}}^{t},\,{X}_{\mathrm{ac}}^{2000}\right)$$

4 $${\mathrm{TAC}}^{t}\,=\,{\mathrm{TAC}}_{\mathrm{obs}}^{t}\,-\,\left.{\mathrm{TAC}}^{t}\,\right|\,{X}_{\mathrm{ac}}$$

After obtaining enhanced TAC for each spatial pixel, we averaged them by total observations from the forest plot. This allowed us to directly associate TAC values from specific forested areas with hydraulic trait data from the same regions, synchronizing the rolling satellite observation windows with the timing of the in situ field campaigns (Extended Data Fig. 2).

### Correlations between TAC and hydraulic traits

To investigate the relationship between TAC and HSM, we employed a two-step analytical approach. First, to test the direct relationship, we fitted a simple linear regression model using 1-TAC as the response variable and HSM as the sole predictor. In this framework, negative TAC values were retained, placing them at the highest end of the resilience spectrum (1-TAC >1). We acknowledge that while negative TAC may reflect rapid biological recovery, it can also be influenced by high-frequency noise^42,76^; determining the exact ecological significance of negative TAC values remains an important area for future research. For each vegetation index, compositing interval and rolling window combination, we computed the adjusted coefficient of determination (*R*^2^), Pearson correlation coefficient (*r*) and corresponding *P* value to evaluate the strength and significance of the associations. These statistics allowed us to compare how different factors influence the sensitivity of TAC to HSM across forest types. Stronger positive correlations were interpreted as more reliable reflections for drought resilience, whereas higher 1-TAC values were associated with lower hydraulic risks.

Second, to evaluate the influence of background water deficit on the correlation relationship, we built multiple regression models, in which CWD was added as an additional predictor alongside HSM. CWD represents the long-term difference between annual evapotranspiration and precipitation. This additive framework allowed us to test whether long-term hydrological conditions modulate the strength of the resistance–resilience relationship beyond what is explained by HSM alone. Model performance was assessed using both adjusted *R*^2^ and Akaike information criterion (AIC), enabling direct comparison with the simple regression models. In cases where the multiple regression model provided a lower AIC, the variance in 1-TAC was attributed to HSM and CWD, highlighting the extent to which background water availability contributes to explaining spatial differences in drought resilience across sites.

### Reporting summary

Further information on research design is available in the Nature Portfolio Reporting Summary linked to this article.

## Supplementary information

Reporting Summary

Peer Review File

## Data Availability

The raw data of the hydraulic traits came from the pan-Amazonian hydraulic traits dataset^49^, which is deposited as a ForestPlots.net data package: https://doi.org/10.5521/forestplots.net/2023_1 (ref. ^97^). This dataset combines newly collected samples from Southern and Western Amazon together with published and unpublished data from Central–Eastern Amazon (refs. ^55,56^ and Jancoski et al., unpublished). We recompiled this dataset as Extended Data Table 1. The climate data used in this study are available from the ERA5-Land reanalysis product (https://cds.climate.copernicus.eu/datasets/reanalysis-era5-land-monthly-means?tab=overview). The forest cover map was accessed from the Global 2010 Tree Cover (30 m) (https://glad.umd.edu/dataset/global-2010-tree-cover-30-m). The Landsat BRDF-corrected surface reflectance data and plant hydraulic data are available via GitHub at https://github.com/GERSL/TAC-HSM.

## Extended data

**Extended Data Table 1:** Plot-level hydraulic traits used in this study

ID	Name	Lon	Lat	MAT (°C)	MAP (mm)	CWD (mm/year)	SampleYear of Pmin	SampleMonth of Pmin	PlotArea (ha)	N	Total sampled basal area in terms of HSM (%)	P50 (MPa)	Pmin (MPa)	HSM (MPa)	Source	P50 measured or compiled?	P50 original source
1	ALP2	-73.44	-3.95000028	25.4	2634	-1328	2017	10	0.44	11	27.32	-1.44	-0.71	0.72	Supplementary Table 2 (ref. ^49^)	measured	
2	ALP1	-73.429998	-3.95000531	25.4	2616	-1316	2017	10	0.48	14	27.16	-1.97	-0.96	1.02	Supplementary Table 2 (ref. ^49^)	measured	
3	CAX	-51.450001	-1.70999799	26.46	2250	-1052	2016	10	0.81	18	25.5	-2.18	-2.51	-0.32	Supplementary Table 2 (ref. ^49^)	compiled	(ref. ^55^)
4	FEC	-67.616697	-10.0667047	25.33	1845	-651	2017	8	1	19	59.43	-2.26	-2.62	-0.32	Supplementary Table 2 (ref. ^49^)	measured	
5	MAN	-60.199997	-2.59999931	25.84	2935	-1516	2016	8	1	17	13.69	-2.07	-1.48	0.59	Supplementary Table 2 (ref. ^49^)	compiled	(ref. ^56^)
6	TAM	-69.300002	-12.7999954	24.76	2695	-1338	2017	9	1	26	59.36	-1.54	-1.52	-0.01	Supplementary Table 2 (ref. ^49^)	measured	
7	NVX	-52.167998	-14.8330001	26.45	1418	-373	2016	8	0.6	7	51.38	-2.95	-3.95	-1	Supplementary Table 2 (ref. ^49^)	compiled	Jancoski et al. (unplublished)
8	SUC	-72.9	-3.29999504	25.39	2965	-1609	2017	11	1	23	23.90	-1.29	-0.97	0.32	Supplementary Table 2 (ref. ^49^)	measured	
9	KEN1	-62.729998	-16.0199992	23.46	1591	-316	2017	8	1	11	75.16	-2.44	-2.1	0.34	Supplementary Table 2 (ref. ^49^)	measured	

Columns from left to right: site included in the study (ID), names of the sites (Name), longitude (Lon) and latitude (Lat) in decimal degrees of community locations, mean annual temperature (MAT; °C), mean annual precipitation (MAP; mm), cumulative water deficit (CWD; calculated by the long-term average actual evapotranspiration - precipitation; mm/year), sample year of Psi_min (SampleYear), sample month of Psi_min (SampleMonth), size of forest plot (PlotArea; ha), the number of tree species measured at each site (N), Total sampled basal area in terms of HSM (%), site-level mean xylem water potential at 50% embolism (P50; MPa), site-level mean midday leaf water potential in the dry season (Pmin; MPa), site-level mean hydraulic safety margins with respect to P50 (HSM; MPa), reference (authors and year), method used to obtain P50 (P50 measured or compiled), and the original reference of P50 (P50 original source). The spatial distribution of these plots is shown in Extended Data Fig. 1.

## Code availability

The custom code used to reproduce all results in this study, developed in MATLAB R2023b and Python 3.11, is available via Zenodo at https://doi.org/10.5281/zenodo.19907314 (ref. ^98^).
