# Supplementary material for: Physiological fidelity of a satellite-derived forest resilience indicator in the Amazon
Source: Nat Ecol Evol. 2026 Jul 21;10(9):1682–93. doi: 10.1038/s41559-026-03116-z (PMC13541618; doi:10.1038/s41559-026-03116-z)
Supplement: Supplementary file 1 [file 41559_2026_3116_MOESM1_ESM.pdf]

nature portfolio

Corresponding author(s): Zhe Zhu and Kexin Song

Last updated by author(s): Apr 27, 2026

Reporting Summary

Nature Portfolio wishes to improve the reproducibility of the work that we publish. This form provides structure for consistency and transparency in reporting. For further information on Nature Portfolio policies, see our Editorial Policies and the Editorial Policy Checklist.

Statistics

For all statistical analyses, confirm that the following items are present in the figure legend, table legend, main text, or Methods section.

n/a	Confirmed	
☐	☒	The exact sample size (n) for each experimental group/condition, given as a discrete number and unit of measurement
☐	☒	A statement on whether measurements were taken from distinct samples or whether the same sample was measured repeatedly
☐	☒	The statistical test(s) used AND whether they are one- or two-sided<br>*Only common tests should be described solely by name; describe more complex techniques in the Methods section.*
☐	☒	A description of all covariates tested
☐	☒	A description of any assumptions or corrections, such as tests of normality and adjustment for multiple comparisons
☐	☒	A full description of the statistical parameters including central tendency (e.g. means) or other basic estimates (e.g. regression coefficient) AND variation (e.g. standard deviation) or associated estimates of uncertainty (e.g. confidence intervals)
☐	☒	For null hypothesis testing, the test statistic (e.g. F, t, r) with confidence intervals, effect sizes, degrees of freedom and P value noted<br>*Give P values as exact values whenever suitable.*
☒	☐	For Bayesian analysis, information on the choice of priors and Markov chain Monte Carlo settings
☒	☐	For hierarchical and complex designs, identification of the appropriate level for tests and full reporting of outcomes
☐	☒	Estimates of effect sizes (e.g. Cohen's d, Pearson's r), indicating how they were calculated

Our web collection on statistics for biologists contains articles on many of the points above.

Software and code

Policy information about availability of computer code

Data collection	Python 3.11 was used to access Landsat surface reflectance time series via the Google Earth Engine Python API, earthengine-api 1.7.22. MATLAB R2023b was used to download the forest cover map for the study area from the University of Maryland Global Land Analysis and Discovery (GLAD) dataset (https://gladxfer.umd.edu/Potapov/TCC_2010/). ERA5-Land reanalysis was accessed directly from the Copernicus Climate Data Store without the use of software tools.
Data analysis	MATLAB R2023b was used for all major data-processing steps, including forest cover extraction, climate variable preparation, satellite time-series analysis (change detection and temporal autocorrelation), random forest regression to remove climate autocorrelation effects in the resilience indicator, and correlation analysis between the resilience indicator and resistance metrics. Python 3.11 was used for conducting Bidirectional Reflectance Distribution Function normalization of surface reflectance time series. Codes are available at GitHub: https://github.com/GERSL/TAC-HSM.

For manuscripts utilizing custom algorithms or software that are central to the research but not yet described in published literature, software must be made available to editors and reviewers. We strongly encourage code deposition in a community repository (e.g. GitHub). See the Nature Portfolio guidelines for submitting code & software for further information.

Data

Policy information about availability of data

All manuscripts must include a data availability statement. This statement should provide the following information, where applicable:

- Accession codes, unique identifiers, or web links for publicly available datasets
- A description of any restrictions on data availability
- For clinical datasets or third party data, please ensure that the statement adheres to our policy

Our analysis relies on publicly available satellite and climate data, and a comprehensive compilation of previously published, in-situ hydraulic trait measurements. No new field data was collected for this study. The raw hydraulic traits compiled from previous publications can be found in the Extended Data Table 1. The climate data used in this study is available from the ERA5-Land reanalysis product (DOI: 10.24381/cds.68d2bb30). The forest cover map was accessed from the Global 2010 Tree Cover (30 m) (https://glad.umd.edu/dataset/global-2010-tree-cover-30-m). The Landsat surface reflectance data were accessed from Google Earth Engine.

Research involving human participants, their data, or biological material

Policy information about studies with human participants or human data. See also policy information about sex, gender (identity/presentation), and sexual orientation and race, ethnicity and racism.

Reporting on sex and gender	N/A
Reporting on race, ethnicity, or other socially relevant groupings	N/A
Population characteristics	N/A
Recruitment	N/A
Ethics oversight	N/A

Note that full information on the approval of the study protocol must also be provided in the manuscript.

Field-specific reporting

Please select the one below that is the best fit for your research. If you are not sure, read the appropriate sections before making your selection.

☐ Life sciences ☐ Behavioural & social sciences ☒ Ecological, evolutionary & environmental sciences

For a reference copy of the document with all sections, see nature.com/documents/nr-reporting-summary-flat.pdf

Ecological, evolutionary & environmental sciences study design

All studies must disclose on these points even when the disclosure is negative.

Study description	We investigated the relationship between an ecological resilience indicator (temporal autocorrelation of satellite-derived vegetation index) and a physiological resistance metric (hydraulic safety margins) across the Amazon forest. We also evaluated how methodological choices, including the selection of vegetation indices, satellite observation frequency, and rolling-window lengths, influence the strength and stability of this correlation.
Research sample	We used 10,000 simple random samples across the entire region (30°S–10°N, 90°W–30°W) for satellite data harmonization and extracted 2,271 stable forest samples for training random forest models which were then applied for factoring out climate autocorrelation effects in the resilience indicator. The spatial resolution of satellite data is 30 m. Hydraulic trait dataset comprises 114 tree species measured across 9 Amazonian forest plots with plot sizes ranging from 0.44–1 ha. For each plot, satellite metrics were aggregated over the corresponding footprint to enable direct comparison between the resilience and resistance metric.
Sampling strategy	Simple random sampling was used to select pixels for satellite sensor-to-sensor harmonization across the study region. For training the random forest regression models, we then identified stable forest samples located within a 500-km radius of each in-situ forest plot, ensuring that the training data reflected the environmental conditions represented by the plot network.
Data collection	All hydraulic trait data were compiled from the peer-reviewed literature cited in Extended Data Table 1. All remote sensing and climate model reanalysis data were accessed from their respective public archives. Kexin Song downloaded and processed all datasets.
Timing and spatial scale	The Landsat data has a spatial resolution of 30 m with a revisit interval of approximately 8 days, covering the period 2000–2024.<br>The climate variables from ERA5-Land reanalysis data are provided at monthly intervals with a spatial resolution of 0.1° (≈9 km).<br>The forest cover information was derived from the Global 2010 Tree Cover product at 30 m resolution.<br>Hydraulic trait measurements were compiled from previously published field studies conducted between 2016 and 2017.

Data exclusions	The TAP forest site (Tavares et al 2023) was excluded due to insufficient Landsat observations during the temporal window required for the core regression analysis. The KEN2 site (Tavares et al 2023) was excluded because of inconsistencies in reported forest cover among studies, which could potentially bias the estimated relationship between resilience and resistance. Within the satellite time series, individual pixels flagged as disturbed by the COLD algorithm were excluded from the "Enhanced TAC" calculation. Furthermore, any data pairs with more than one consecutive missing observations after temporal compositing were also excluded to avoid artifacts from excessive interpolation.
Reproducibility	All custom code used for analysis is publicly available and has been successfully run to replicate the findings presented in the manuscript.
Randomization	Randomization is an integral part of our machine learning workflow. The random forest models used for our "Enhanced TAC" calculation rely on bootstrapping (random sampling with replacement) to create the training data for each tree. To ensure that our model training is fully reproducible, we set a fixed random seed in our MATLAB scripts prior to executing the regression.
Blinding	Blinding is not relevant to this study as it is a computational analysis of pre-existing datasets and does not involve experimental groups or participants.

Did the study involve field work? ☐ Yes ☒ No

Reporting for specific materials, systems and methods

We require information from authors about some types of materials, experimental systems and methods used in many studies. Here, indicate whether each material, system or method listed is relevant to your study. If you are not sure if a list item applies to your research, read the appropriate section before selecting a response.

Materials & experimental systems

n/a	Involved in the study
☒	☐ Antibodies
☒	☐ Eukaryotic cell lines
☒	☐ Palaeontology and archaeology
☒	☐ Animals and other organisms
☒	☐ Clinical data
☒	☐ Dual use research of concern
☒	☐ Plants

Methods

n/a	Involved in the study
☒	☐ ChIP-seq
☒	☐ Flow cytometry
☒	☐ MRI-based neuroimaging

Plants

Seed stocks	N/A
Novel plant genotypes	N/A
Authentication	N/A
