# Supplementary material for: Physiological fidelity of a satellite-derived forest resilience indicator in the Amazon
Source: Nat Ecol Evol. 2026 Jul 21;10(9):1682–93. doi: 10.1038/s41559-026-03116-z (PMC13541618; doi:10.1038/s41559-026-03116-z)
Supplement: Supplementary file 2 [file 41559_2026_3116_MOESM2_ESM.pdf]

nature portfolio

Peer Review File

Physiological fidelity of a satellite-derived forest resilience indicator in the Amazon

Corresponding Author: Dr Zhe Zhu

Version 0:

Decision Letter:

5th December 2025

*Please ensure you delete the link to your author homepage in this e-mail if you wish to forward it to your co-authors.

Dear Dr Zhu,

Thank you for your patience while your manuscript entitled "The Physiological Key to a Satellite-derived Forest Resilience Indicator" was under review. It has now been assessed by three reviewers, whose reports are provided below. As you will see, the reviewers find the work of interest but have raised a number of concerns which will need to be addressed before we can offer publication in Nature Ecology & Evolution. We would therefore like to see your responses to their criticisms along with a revised manuscript.

We are committed to providing a fair and constructive peer-review process. Do not hesitate to contact us if there are specific requests from the reviewers that you believe are technically impossible or unlikely to yield a meaningful outcome.

When revising your manuscript:

* Please provide the manuscript in Microsoft Word format and highlight all changes in the same file or a copy.

* Include a “Response to reviewers” document detailing, point-by-point, how you addressed each reviewer comment. If no action was taken to address a point, you must provide a compelling argument. This response will be sent back to the reviewers along with the revised manuscript.

* If you have not done so already please begin to revise your manuscript so that it conforms to our Article format instructions at http://www.nature.com/natecolevol/info/final-submission. Refer also to any guidelines provided in this letter.

* Include a revised version of any required reporting checklist. It will be available to referees (and, potentially, statisticians) to aid in their evaluation if the manuscript goes back for peer review. A revised checklist is essential for re-review of the paper.

* Extended Data Figures - please ensure that any supplementary figures and tables that are crucial to the manuscript’s conclusions are converted into Extended Data figures and tables to increase visibility of these data. Extended Data figures and tables are online-only (present in the online PDF and full-text HTML versions of the paper), peer-reviewed display items that provide essential background to the article but are not included in the main article due to space constraints. A maximum of ten Extended Data display items (figures and tables) is permitted.

We hope to receive your revised manuscript within three months. If you cannot send it within this time, please let us know. We will be happy to consider your revision so long as nothing similar has been accepted for publication at Nature Ecology & Evolution or published elsewhere. Once ready, please use the link below to submit your revised manuscript and related files:

Link Redacted

Note: This URL links to your confidential home page and associated information about manuscripts you may have submitted, or that you are reviewing for us. If you wish to forward this email to co-authors, please delete the link to your homepage.

Nature Ecology & Evolution is committed to improving transparency in authorship. As part of our efforts in this direction, we

are now requesting that all authors identified as ‘corresponding author’ on published papers create and link their Open Researcher and Contributor Identifier (ORCID) with their account on the Manuscript Tracking System (MTS), prior to acceptance. ORCID helps the scientific community achieve unambiguous attribution of all scholarly contributions. You can create and link your ORCID from the home page of the MTS by clicking on ‘Modify my Springer Nature account’. For more information please visit please visit www.springernature.com/orcid.

We look forward to seeing the revised manuscript.

[redacted]

****

Reviewers' comments:

Reviewer #1 (Remarks to the Author):

The physiological key to a satellite-derived forest resilience indicator

The authors ask the primary question of whether satellite-observed TAC truly reflects forest resilience, or is it a statistical artifact? Lag-1 temporal autocorrelation (TAC) variation closely resembles the trajectory of ecosystems in response to perturbations. Depending on the ecosystem vulnerability in the past and the system takes longer to recover (beyond a tipping point). The authors found a significant association between TAC estimates and site hydraulic safety margins and claim a strong a mechanistic link. The mechanism here was not a clearly investigated. Improvements should be may to explaining the hydraulic basis for variation in TAC and more evidence for resilience and resistance behaviors should be used to demonstrate TAC's capacity.

Comments
Text:

line 84-86: Please provide reference.

line 85: No need for p88 notion if it is not used in the text.

line 87-89: The goal of the paper, so far seems to be to evaluate the mechanisms underlying the use of TAC why wouldn't you track the changes in TAC from a well hydrated stated to an extreme drought to understand the bounds of its utility?

line 92-93: Suggest using a clearer example i.e., partial embolism of what xylem or what hydraulic conditions.

line 142: Both "TAC" and "1-TAC" are used throughout the text. I suggest maintaining 1-TAC throughout the text for clarity and instead of "resilience proxy" since TAC is the primary variable of interest here.

line 142: Additionally, HSM_p50 is confusing because it conflates two distinct traits. HSM here was only linked to P50 so it doesn't add more information for the reader.

line 145: The relationship between these sets of variables in Figure 2

line 143: Suggest replacing proxy with proxies.

line 147: It is not clear where the mechanistic implications come from here. Figure 2 shows that these two quantities are related alone.

line 148: This paragraph also should address why more sites are showing HSMs greater than 0. Similarly, "mechanistic" does not seem appropriate here. HSM is not change because of TAC. For example, mortality would be a better supplementary variable here to show that forests with greater HSM didn't experience hydraulically driven mortality compared to the others.

line 153: Suggest replacing "foundational link". It oversells what is mentioned in the previous paragraph.

line 166: Can you elaborate here on what it means for hydraulic resistance and resilience to become decoupled with a trait-based example please?

line 169: In Figure 2, There are many points outside of the confidence bands of your model (e.g., "MAN"). Are these also upland sites? Suggest excluding this conclusion

line 173: Please define "site-years" here

line 495-496: Please specify what organs were used for each metric. Were only shoot or branch Pmin and P50 used? When

during the season were measurements taken? If the values were determined earlier in the season you would have positive HSM given a less negative Pmin compared to mid-season.

line 496-498: Please provide the range of mean annual precipitation and temperature to characterize how widely they differ.

line 489: For the final estimates of Pmin, was leaf or xylem water potential used? They can be significantly different with enough tissue capacitance.

line 501: Species level traits is not necessarily a bad criterion of hydraulic data. Were the traits not weighted by species composition before your analysis?

line 504: what were the "same methods" used for measuring hydraulic traits? How representative of the population were the species for which you found hydraulic data?

line 505: Did these studies measure a representative number of individuals for each species in the plots?

line 569: Were the hydraulic trait measurements taken at the same time? What time during the season were there measured? If P50 was measured on younger tissues your measurements would underestimate the drought tolerance.

Figures:

Figure 3: Suggest remove panel a. It seems unnecessary if no correlations were possible. Additionally, the importance of bimonthly vs quarterly relationships needs to be discussed in the introduction.

Figure 2 caption needs better description of the satellites and indicated that those are satellites in the caption.

Extended Data figures 1 and 2 do not add to your story. The audience is highly likely to understand how prevalent the research is.

Extended Data Table 1 needs references for the "What does it measure" column

Reviewer #2 (Remarks to the Author):

Review – Song et al. – Nature E&E
The paper by Song et al. makes an explicit link between the widely used resilience indicator temporal autocorrelation and a physiological plant response to water deficits, based on a number of plot locations in the Amazon. I think that the general idea of the paper is sound, but there needs to be some improvement around the methodological treatment of the remote sensing data, as well as in the framing of the paper. The authors claim that they find a general relationship, but this is based on a few stations (covering mostly one month of data each) in a confined geographic location – this is not at all general to global-scale analyses, or to how resilience may or may not be changing through time. The data limitations and caveats need to be made more explicit, as well as the generality of the results. I do not doubt that there is a link between hydrologic sensitivity and temporal autocorrelation, but I am not completely convinced the methods used here are substantial enough for the claims of the authors. I leave some more specific comments below.

Title – I recommend modifying this to be less general – this is a relationship that was found for a gradient in the Amazon, not a global relationship. I think the work is interesting, but the title (and abstract) should be more explicit about what you actually study: a link between hydrologic safety margins and TAC in the Amazon over a small number of stations.
Line 65-67 – This explicit link between real recoveries after a disturbance and the theoretical indicators AC1 and variance was made by Smith et al. 2022, Nature Climate Change (https://www.nature.com/articles/s41558-022-01352-2).
Line 68-69 – Recent work has made this reliance on data fidelity more explicit: https://arxiv.org/abs/2505.19034
Figure 3 and Lines 310-314 - I think this is a very nice analysis – but would be very specific to the sites chosen. The optimal time sampling and moving windows would be potentially very different for different types of vegetation (e.g., grasslands grow much faster than forests). The specific application here where the sites are all in somewhat similar vegetation types should be sound, but it should be emphasized that these ideal spacings and windows are not universal, and would also vary substantially with e.g. spatial resolution. More plot-scale sites in different vegetation communities would be needed to test how universal this relationship is.
Line 543 – Did you try using single-sensor time series instead? Resilience estimates like TAC can be heavily biased by changes in instrumentation (https://esd.copernicus.org/articles/14/173/2023/, https://www.nature.com/articles/s41558-025-02328-8), so it would be worth making sure that doesn't bias your TAC too much. Each plot is only over a single year, so you could calculate a TAC for just that year, for example. Alternatively, to keep with your previous framework, you could get the TAC in rolling windows based on individual sensors. You could then feed that forward into your regression, having one TAC value per satellite. As a second note, there is not much discussion of the time period you used – the data from the plots is mostly a single year around 2015 sampled in a single month. The comparison to long-term TAC needs to be better justified, as well as the explicit choices made for the time periods of the satellite data you used.
Line 550 – Landsat 7 should be excluded much earlier – there is serious drift in the time of day of data collection: https://tc.copernicus.org/articles/18/5259/2024/, Figure 2.
Section starting at 568 – Do you fit your harmonic models over all Landsat data for the long period, and then only calculate

TAC on the year corresponding to the field data? Or do you use a long-term TAC as a comparison? This is not clear, and could lead to very different results. The irregular and overlapping data sampling is one potential issue of combining sensors, and the comparison of a long-term TAC (from Landsat 5 forward means from the 80s) to a single plot-year is a bit of a reach. The amount of data used for the various different steps in the MS needs to be made much clearer.

Reviewer #2 (Remarks on code availability):

I was not able to find any of the python scripts in the Github (they are listed in the code repository, but are not available on the Github), so cannot comment on the Earth Engine processing chain. I am not proficient in Matlab so cannot comment on the application of the main codes of the MS.

Reviewer #3 (Remarks to the Author):

Overall, the manuscript presents a valuable and timely contribution to understanding forest resilience through remote sensing. However, several aspects require attention to strengthen its scientific rigor and contextual accuracy:
1. Involvement of Original Data Contributors
The study relies heavily on Brazilian field measurements and previously published hydraulic trait datasets. Authors of these original studies should be involved as co-authors or acknowledged collaborators to ensure proper attribution, compliance with local research permits, and accurate ecological interpretation.

2. Contextualizing Novelty Claims
While the insights on TAC and its physiological link are important, claims of being the "first mechanistic investigation" should be moderated. TAC has been mechanistically explored in other domains (e.g., aquatic ecosystems, microbial systems, neuroscience, climate modeling). The true novelty here lies in the field-based physiological validation for forests using hydraulic traits and the methodological guidance for remote sensing applications.

3. Strength of Remote Sensing Insights
The discussion on observation frequency, rolling window size, and vegetation index selection is a major strength of the paper. These methodological clarifications are highly valuable for improving TAC-based resilience monitoring.

4. Compositing and Gap-Filling Methods
More transparency is needed regarding the compositing approach and gap-filling procedures described in Extended Data Fig. 6. Gap-filling can introduce bias in TAC estimates, and its impact should be explicitly evaluated. Ideally, the authors should either avoid gap-filling or quantify its effect on TAC calculations to ensure robustness.

In more detail explained:

1. Given that the validation relies on Brazilian in-situ hydraulic trait datasets collected under local permits, the paper would benefit from the inclusion of Brazilian collaborators (e.g., investigators from INPA/UNICAMP or original dataset authors) as co-authors or acknowledged contributors. This will help ensure accurate ecological interpretation of plot contexts (upland vs. floodplain) and transparent reporting of permits (SISBIO) and data licenses (e.g., CC BY-SA via ForestPlots).

2. The claim that this study provides the first mechanistic investigation of TAC should be qualified. Temporal autocorrelation has been mechanistically linked to resilience in multiple domains beyond terrestrial forests. For example, Shinn et al. (Nature Neuroscience, 2023) demonstrated that TAC explains functional brain network topology and state transitions, highlighting its role in recovery dynamics in neural systems. Similarly, Lenton et al. (Nature Communications, 2023) applied TAC as an early-warning metric for climate tipping points in Earth system models, and Batt et al. (PLOS ONE, 2019) used TAC to detect resilience loss in lake ecosystems approaching eutrophication. These studies show that TAC's mechanistic interpretation as an indicator of slowing recovery is well established across disciplines; the novelty here lies in the field-based physiological validation for forests using hydraulic traits, not in being the first mechanistic study overall.
Suggested references to include:
Shinn M. et al. Functional brain networks reflect spatial and temporal autocorrelation. Nature Neuroscience 26, 867–878 (2023).
Lenton T. M. et al. Early warning of climate tipping points from autocorrelation signals. Nature Communications 14, 498 (2023).
Batt R. D. et al. Detecting critical slowing down in lakes approaching eutrophication. PLOS ONE 14, e0216362 (2019).

Overview of existing studies and results already published to clarify the novelty of the current manuscript. The following studies should be properly cited and included in the current manuscript:

Mechanistic Underpinnings of TAC
• Physiological Link: TAC is mechanistically linked to plant hydraulic safety margin (HSM_P50), a key trait for drought resistance. Field data from tropical forests show a strong correlation between HSM_P50 and the resilience proxy (1-TAC), indicating that community-level drought resistance constrains forest recovery speed and resilience (Tavares et al., 2023; Oliveira et al., 2021).
• Critical Slowing Down: Theoretical and empirical studies confirm that as ecosystems approach critical thresholds (e.g., due

to drought), TAC increases—a phenomenon known as "critical slowing down"—signaling reduced resilience and higher risk of abrupt transitions (Smith et al., 2022; Forzieri et al., 2022; Smith & Boers, 2023; Zhang et al., 2025; Wu et al., 2024).

Methodological Reliability and Limitations

• Influence of Water Availability: TAC-based resilience is strongly modulated by background water availability and cumulative water deficit, with models incorporating both explaining up to 62% of resilience variance (Smith & Boers, 2023; Tavares et al., 2023; Peters et al., 2021).
• Vegetation Index and Temporal Resolution: The reliability of TAC as a resilience indicator depends on the choice of vegetation index, temporal frequency of satellite observations, and rolling window size. High-biomass regions (e.g., dense tropical forests) present challenges for accurate TAC-based resilience estimation (Smith & Boers, 2023; Müller et al., 2024; Smith et al., 2023).
• Fragmentation and Biome Differences: The relationship between TAC and resilience varies by biome and is influenced by landscape fragmentation, with different patterns observed in tropical, temperate, and boreal forests (Su et al., 2025; Forzieri et al., 2022; Lv et al., 2025).

References

Su, Y. et al. (2025). Pervasive but biome-dependent relationship between fragmentation and resilience in forests. Nat. Ecol. Evol.
Smith, T., Traxl, D., & Boers, N. (2022). Empirical evidence for recent global shifts in vegetation resilience. Nat. Clim. Change, 12, 477–484.
Forzieri, G. et al. (2022). Emerging signals of declining forest resilience under climate change. Nature, 608, 534–539.
Smith, T., & Boers, N. (2023). Global vegetation resilience linked to water availability and variability. Nat. Commun., 14.
Smith, T., & Boers, N. (2023). Reliability of vegetation resilience estimates depends on biomass density. Nat. Ecol. Evol., 7, 1799–1808.
Müller, M. et al. (2024). Response and resilience to drought in northern forests revealed by Sentinel-2. Int. J. Remote Sens., 45, 5130–5157.
Tavares, J. et al. (2023). Basin-wide variation in tree hydraulic safety margins predicts the carbon balance of Amazon forests. Nature, 617, 111–117.
Zhang, L. et al. (2025). Remote sensing and critical slowing down modeling reveal vegetation resilience in the Three Gorges Reservoir Area, China. Remote Sens.
Wu, X. et al. (2024). Declining resilience of forest carbon sinks linked to increased atmospheric water deficit during droughts in the Northern Hemisphere. Environ. Res. Lett., 20.
Lv, X. et al. (2025). Exploring the resilience of global vegetation ecosystem: Nonlinearity, driving forces, and management. J. Environ. Manag., 377, 124634.
Oliveira, R. et al. (2021). Linking plant hydraulics and the fast-slow continuum to understand resilience to drought in tropical ecosystems. New Phytol.
Peters, J. et al. (2021). Living on the edge: A continental-scale assessment of forest vulnerability to drought. Glob. Change Biol., 27.
Smith, T. et al. (2023). Reliability of resilience estimation based on multi-instrument time series. Earth Syst. Dyn.

Reviewer #3 (Remarks on code availability):

The public release of the TAC-HSM code is a strong step toward transparency and reproducibility. The repository appears to contain MATLAB/Python scripts for TAC computation, Landsat harmonization, RF models to isolate climate autocorrelation, and plotting/analysis workflows (folders such as TAC, L57_L89_Harmonization, RFmodel, and scripts like runTAC_Landsat_allSample.m, obtainEnhancedTAC_FieldSample.m). However, based on the GitHub page, I cannot assess execution quality, test coverage, or long-term maintenance. The repo shows only a handful of commits and no formal releases or recent updates, suggesting it is currently static. [github.com], [github.com]
Actionable suggestions (minimal burden, high impact):

Add a Reproducibility README with a one-command run
Include exact software versions (MATLAB 2023b, Python 3.10), package lists, OS details, and a small sample dataset so that users can run an end-to-end test in minutes. Provide expected outputs (e.g., test TAC values, R^2) for verification. [github.com]
Environment files & containers
Publish a requirements.txt/environment.yml for Python, and (if feasible) a Dockerfile or conda environment to standardize dependencies. A simple container greatly increases reproducibility for long-lived projects. [github.com]
Versioning & releases
Tag a v1.0 GitHub Release with notes describing what's included, what's tested, and the figures/results reproduced. Add semantic versioning for future updates. Currently, there are no releases. [github.com]
Basic tests
Provide small unit/regression tests (e.g., TAC on synthetic signals; harmonization checks; RF model determinism with fixed seeds). Even a minimal CI (GitHub Actions) that runs these tests signals ongoing maintenance. [github.com]
Data availability pointers
If large inputs cannot be tracked, add README_data.md with links and checksums (e.g., ForestPlots/Tavares tables, ERA5-Land pointers) and a script to fetch/prepare small subsets to validate the pipeline. [github.com]
Repository housekeeping

Replace .asv autosave files with stable .m scripts to avoid confusion (runTAC_Landsat_allSample.asv, etc.).

Add a license (e.g., MIT) and a CONTRIBUTING.md to clarify reuse and maintenance plans. [github.com]

Version 1:

Decision Letter:

7th April 2026

Dear Zhe,

Thank you for submitting your revised manuscript "Physiological fidelity of a satellite-derived forest resilience indicator in the Amazon" (NATECOLEVOL-25103871A). It has now been seen again by two of the original reviewers, whose comments you will find below. On the basis of their feedback, we will be happy in principle to publish it in Nature Ecology & Evolution, pending minor revisions to satisfy the reviewers' final requests and to comply with our editorial and formatting guidelines.

We are now performing detailed checks on your paper and will send you a checklist detailing our editorial and formatting requirements in about a week. Please do not upload the final materials and make any revisions until you receive this additional information from us.

Thank you again for your interest in Nature Ecology & Evolution. Please do not hesitate to contact me if you have any questions.

[redacted]

Reviewer #1 (Remarks to the Author):

Thank you for your thorough responses to my comments. Your work is exciting and novel--merging plant physiology and remote sensing.

I have a few minor comments:

line 81: Missing an "s" in assess
line 250: Suggest to replace "calculated" with "represented"
line 252-253: Suggest to replace "integrates embolism resistance to actual plant operation" to ""
line 260-61: Suggest to replace "season decreases in water availability"

Reviewer #1 (Remarks on code availability):

For clarity to future readers it may be useful to remove folders and files that not involved in the analysis. I appreciate the .txt instructions but there are many files that are not sourced.

Reviewer #2 (Remarks to the Author):

Review – Song et al. – Nature E&E – Revision 1

I would like to thank the authors for their thorough Revision – the majority of my original comments were addressed. I have two remaining questions about the ED figures:

ED3 – Negative TAC? That implies high is always followed by low, so there is no recovery process. How do you interpret that in your framework? In other work negative TAC is masked as data noise.

ED4 - Gap filling clearly biases autocorrelation high. How does that propagate through into your results? You could compare to nan-aware TAC (e.g., np.ma.corrcoeff()) to make sure that isn't a strong factor in your results.

Reviewer #2 (Remarks on code availability):

The repository is much improved from the previous version, and the Earth Engine codes look fine to me.

Open Access This Peer Review File is licensed under a Creative Commons Attribution 4.0 International License, which permits use, sharing, adaptation, distribution and reproduction in any medium or format, as long as you give appropriate credit to the original author(s) and the source, provide a link to the Creative Commons license, and indicate if changes were made.
In cases where reviewers are anonymous, credit should be given to 'Anonymous Referee' and the source.
The images or other third party material in this Peer Review File are included in the article's Creative Commons license, unless indicated otherwise in a credit line to the material. If material is not included in the article's Creative Commons license and your intended use is not permitted by statutory regulation or exceeds the permitted use, you will need to obtain permission directly from the copyright holder.
To view a copy of this license, visit https://creativecommons.org/licenses/by/4.0/

Replies to editor and reviewers

We gratefully thank all reviewers for their time spent making their constructive remarks and useful suggestions, which have helped us to substantially improve the quality of the manuscript. Each suggested revision and comment brought forward by the reviewers was incorporated and considered. The comments of the reviewers (in black) are responded to point by point below (in **blue**), and the revisions are indicated in ***green***. Below are the major revisions implemented in this updated manuscript:

1. Included as a co-author the scientist who conducted the in-field hydraulic trait measurements to enhance accurate ecological interpretation of the field data.
2. Clarified the definition and interpretation of hydraulic safety margin (HSM). We highlighted that the definition of HSM as used in this study is time-specific and context-dependent. We articulated its mechanistic linkage to embolism, hydraulic failure, and forest resilience as indicated by lag-1 temporal autocorrelation (TAC). In addition, we discussed potential factors that may lead to trade-offs between plant drought resistance and resilience.
3. Refined the scope of the study and strengthened discussion on future directions. We clarified that this work provides a mechanistic evaluation of TAC using community-level datasets from the Amazon forests. We explicitly acknowledged data limitations and caveats and outlined future opportunities for integrating field-based measurements and remote sensing observations to advance global resilience assessment.
4. Strengthened analysis robustness and improved methodological transparency. Specifically, we excluded four plots to ensure site consistency, as these plots either used a different P50 measurement method, included flooded forests, or did not report community-weighted mean HSM values. We also conducted sensitivity tests to evaluate the impacts of compositing and gap-filling strategies on the TAC-HSM relationship and further confirmed this relationship using satellite time series from a single sensor. In addition, we updated the source code in GitHub and revised figures accordingly.

Reviewer #1 (Remarks to the Author):

The authors ask the primary question of whether satellite-observed TAC truly reflects forest resilience, or is it a statistical artifact? Lag-1 temporal autocorrelation (TAC) variation closely resembles the trajectory of ecosystems in response to perturbations. Depending on the ecosystem vulnerability in the past and the system takes longer to recover (beyond a tipping point). The authors found a significant association between TAC estimates and site hydraulic safety margins and claim a strong a mechanistic link. The mechanism here was not a clearly investigated. Improvements should be may to explaining the hydraulic basis for variation in TAC and more evidence for resilience and resistance behaviors should be used to demonstrate TAC's capacity.

Response: Thank you. In the revised manuscript, we explicitly articulate the hydraulic pathway linking HSM and TAC. Forests with smaller HSM operate closer to critical embolism thresholds and are therefore more vulnerable to hydraulic failure during drought. Partial xylem embolism reduces hydraulic conductance even if it does not immediately cause mortality. Since embolized vessels are not rapidly restored, reduced conductance constrains carbon sequestration and slows canopy recovery following stress. This delayed recovery increases the persistence of vegetation anomalies through time, resulting in elevated lag-1 TAC.

We also clarify why Amazonian forests provide a particularly valuable model system for testing the physiological basis of TAC. First, it spans strong hydroclimatic gradients, from relatively wet to seasonally water-limited forests, enabling evaluation of resistance–resilience relationships under contrasting environmental conditions. Second, it contains high functional diversity and variation in hydraulic strategies, increasing the likelihood of detecting mechanistic links between plant physiology and satellite-derived TAC. Third, recurrent drought events over the past decades in Amazon forests have heightened concern about forest stability, underscoring the need to understand the physiological foundations of resilience in this area. At present, we do not have sufficiently standardized field hydraulic data to conduct a rigorous global-scale analysis, however, we explicitly acknowledge that broader multi-site and multi-disturbance validation would further strengthen evidence for TAC as a general indicator of ecosystem resilience.

To address specific comments, we have conducted additional analyses and revised the manuscript accordingly. We appreciate the reviewer's valuable feedback, which has significantly improved the quality of our manuscript.

Comments

Text:
line 84-86: Please provide reference.

Response: Thank you for the comment. We have included the reference below to clarify the definition of hydraulic safety margin used in this study (**Lines 89-91**): *"The hydraulic safety*

margin (HSM[51]), defined as the distance from the plant water potential during the peak of the dry season (Pmin) to P50, integrates embolism resistance to actual plant operation.”

Reference:

[51] Meinzer, F. C., Johnson, D. M., Lachenbruch, B., McCulloh, K. A., & Woodruff, D. R. (2009). Xylem hydraulic safety margins in woody plants: coordination of stomatal control of xylem tension with hydraulic capacitance. *Functional Ecology*, 23(5), 922-930.

line 85: No need for p88 notion if it is not used in the text.

Response: Thank you for the suggestion. We have removed the p88 notation in the main text and the Methods session.

line 87-89: The goal of the paper, so far seems to be to evaluate the mechanisms underlying the use of TAC why wouldn't you track the changes in TAC from a well hydrated stated to an extreme drought to understand the bounds of its utility?

Reponses: We agree that tracking changes in TAC from a well-hydrated state to an extreme drought is essential to understand the operational bounds of its utility (e.g., assessing whether the indicator loses functionality under extreme water stress). However, addressing this requires two critical steps.

First, before we can confidently analyze temporal changes in TAC, we must prove that the baseline signal actually reflects physiologically meaningful resilience. Without this mechanistic proof, temporal fluctuations in the satellite signal remain difficult to interpret. Second, continuous, day-to-day measurements of community-level plant hydraulics are logistically impossible; such field data only exist as static snapshots. Therefore, to overcome this data limitation, our study design leverages a spatial gradient rather than a temporal one to firmly establish this foundational physiological baseline across different forest plots.

Once this spatial baseline was established, we were able to test the temporal bounds of TAC’s utility, which we demonstrate in Extended Data Fig 3. To directly answer your question regarding the bounds of its utility, this figure tracks the continuous trajectory of TAC through a severe decennial drought. Crucially, we do not just track the satellite index; we anchor it to a known physiological extreme. During the peak of this drought in September 2017, field measurements recorded a community-level HSM of -0.01 MPa. Physiologically, an HSM of -0.01 indicates that the forest had reached the absolute brink of hydraulic failure, experiencing a 50% loss of xylem conductance.

Because the satellite-derived TAC signal successfully tracked the ecosystem’s trajectory right down to this specific biological catastrophe without decoupling or turning into statistical noise, we can confidently conclude that the indicator does not "break" under extreme stress. Instead, this rare

temporal alignment proves that TAC maintains its functional and ecological validity even when the ecosystem is pushed to its absolute physiological bounds. We have updated the main text to explicitly clarify this study design choice and the bounds of the indicator's utility (**Lines 300-312):** *"To test the operational bounds of this indicator prior to such a catastrophic shift, we tracked its continuous trajectory across a severe decennial drought at Tambopata plot three (TAM) (Extended Data Fig 3). Crucially, this satellite time-series is anchored by field data: during the drought peak in September 2017, the forest reached a critical physiological limit (HSM = -0.01 MPa, indicating a 50% loss of xylem conductance). Because the TAC signal successfully tracked the ecosystem right down to the edge of hydraulic failure without decoupling into noise, we demonstrate that the indicator maintains its functionality even at the extreme bounds of physiological tolerance. However, because field-measured hydraulic traits require labor-intensive campaigns, they are currently limited to static snapshots (Extended Data Fig 6), necessitating our use of a spatial gradient to establish this baseline. Fully bounding TAC across different climatic transitions will ultimately require pairing time series of satellite observations with continuous, in-situ physiological monitoring. Evaluating dynamic, temporal changes in TAC as forests transition from well-hydrated states into extreme drought remains an important next step."*

line 92-93: Suggest using a clearer example i.e., partial embolism of what xylem or what hydraulic conditions.

Response: Thank you for the suggestion. We have clarified the corresponding sentence and added appropriate references (**Lines 91 – 105**): *"Partial embolism of plant xylem may reflect a loss of some xylem vessels, that reduces conductance, but not above the threshold that would result in mortality. Embolism that does not result in mortality cannot be reversed in the short term and reduced hydraulic conductance would constrain forest recovery[52]. In this sense, plants operating at low HSM have increased exposure to xylem damage during drought and potentially need high recovery capability, whereas plants with high HSM maintain a larger safety buffer at the cost of maintaining redundant safety margin at most periods[53,54]."*

Reference:

[52] Brodribb, Tim J., and Hervé Cochard. Hydraulic failure defines the recovery and point of death in water-stressed conifers. *Plant physiology* 149.1 (2009): 575-584.

[53] Oliveira, Rafael S., et al. Linking plant hydraulics and the fast–slow continuum to understand resilience to drought in tropical ecosystems. *New Phytologist* 230.3 (2021): 904-923.

[54] B. Eller, Cleiton, et al. Xylem hydraulic safety and construction costs determine tropical tree growth. *Plant, Cell & Environment* 41.3 (2018): 548-562.

line 142: Both "TAC" and "1-TAC" are used throughout the text. I suggest maintaining 1-TAC

throughout the text for clarity and instead of "resilience proxy" since TAC is the primary variable of interest here.

Response: We have revised the manuscript to use 1-TAC, instead of term “resilience proxy”, throughout the text to improve clarity and consistency.

line 142: Additionally, HSM_p50 is confusing because it conflates two distinct traits. HSM here was only linked to P50 so it doesn't add more information for the reader.
Response: Thank you for the helpful comment. We have removed ‘_p50’ from all the HSM mentioned in the manuscript to improve clarity.

line 145: The relationship between these sets of variables in Figure 2.
Response: Thank you. We have linked these sets of variables with their corresponding labels in Fig 2.

line 143: Suggest replacing proxy with proxies.
Response: Thank you for the suggestion. We have corrected this in the revised manuscript.

line 147: It is not clear where the mechanistic implications come from here. Figure 2 shows that these two quantities are related alone.

Response: Thank you for raising this point. We agree that the mechanistic interpretation was not clearly articulated in the previous version. We would like to highlight that the TAC-HSM relationship observed in Fig 2 is consistent with the hypothesized physiological mechanism in which **forest plots with greater drought resistance exhibit higher resilience**. In the revised manuscript, we have reworded the sentence to more accurately reflect this interpretation. The revision reads as follows (**Lines 160 – 162**): *“These results provide the first direct evidence supporting our hypothesis that landscape-scale resilience captured by TAC is mechanistically closely linked to the physiological drought resistance of the constituent trees quantified by HSM.”*

line 148: This paragraph also should address why more sites are showing HSMs greater than 0. Similarly, "mechanistic" does not seem appropriate here. HSM is not change because of TAC. For example, mortality would be a better supplementary variable here to show that forests with greater HSM didn't experience hydraulically driven mortality compared to the others.

Response: Thank you for your constructive comments. We have clarified in the revised manuscript why many sites exhibit positive HSM values. Specifically, at most plots the observed minimum midday leaf water potential (Pmin) did not exceed P50, indicating that hydraulic stress was not severe enough to approach critical hydraulic thresholds during the measurement year.

We also agree that HSM only partially explains the variation in resilience captured by TAC (Fig 2). Accordingly, we have revised the text to avoid implying a causal relationship and instead frame HSM as an independent physiological metric relevant for interpreting TAC patterns.

Regarding the suggestion to include mortality, we agree that sudden increases in tree mortality can result from losses of ecosystem resilience. However, the primary objective of this study is to evaluate whether TAC derived from satellite-derived vegetation indices reliably reflect forest resilience, rather than to assess TAC as an early-warning indicator of mortality. This distinction helps clarify the ecological basis of TAC as a resilience indicator, instead of a direct predictor of mortality events. We have made this clarification explicit in the revised manuscript (**Lines 292-297**): *"In contrast, a long-term increase in TAC at a given location would indicate a loss of resilience, likely reflecting a gradual structural shift driven by accumulating damage, such as irreversible xylem cavitation or low-level mortality, and slow replacement. Crucially, this interpretation also defines the boundaries of what TAC can measure. TAC is an early warning indicator of declining stability within a forest state that may signal elevated mortality risk."*

line 153: Suggest replacing "foundational link". It oversells what is mentioned in the previous paragraph.

Response: Thank you for the comment. We have replaced "foundational link" to "physiological link" to better position the significance of our study.

line 166: Can you elaborate here on what it means for hydraulic resistance and resilience to become decoupled with a trait-based example please?

Response: Thank you for this insightful comment. Conceptually, a trade-off between hydraulic resistance and resilience can occur when the specific traits that enable forests to withstand drought stress differ from those that promote recovery. For example, Hu et al. (2022) showed that drought-resistant forests tend to be dominated by species with conservative hydraulic traits, such as higher HSM and smaller vessel diameters, whereas forests exhibiting high resilience are dominated by species with higher photosynthetic capacity (e.g., higher light-saturated photosynthetic rates), greater water use efficiency (e.g., higher sensitivity of canopy stomatal conductance per unit land area to drought). Rooting depth is another example, whereby plants that invest in deep roots may be resistant to short time-scale drought as they have stable connections to deeper water. However, they may exhibit low resilience as the time scale of the recovery is determined by the hydrological processes that control the refilling of deeper soils (Knighton and Berghuijs, 2023).

However, upon further evaluation of our data, we found that the interpretation of the relationship decoupling in the original paragraph was disproportionately influenced by the ZF-2 plot, which differs from most other sites in both plot characteristics (ZF-2 is a mixed upland and seasonally flooded forest) and the method used to measure P50 (bench dehydration approach). To avoid

potential confusion or overinterpretation driven by these site-specific differences, we have excluded ZF-2 and removed this paragraph from the revised manuscript.

Reference:

Hu, Y., Xiang, W., Schäfer, K. V., Lei, P., Deng, X., Forrester, D. I., ... & Peng, C. (2022). Photosynthetic and hydraulic traits influence forest resistance and resilience to drought stress across different biomes. *Science of the Total Environment*, *828*, 154517.

Knighton, J., & Berghuijs, W. R. (2023). Water ages explain tradeoffs between long-term evapotranspiration and ecosystem drought resilience. *Geophysical Research Letters*, *50*(10), e2023GL103649.

line 169: In Figure 2, There are many points outside of the confidence bands of your model (e.g., "MAN"). Are these also upland sites? Suggest excluding this conclusion.

Response: Thank you for this constructive comment. With the exception of the ATTO and ZF-2 plots, which contain a mixture of upland and seasonally flooded forests, all other plots including MAN used in analysis represent upland sites. We appreciate the reviewer's careful attention to the data. To reduce potential bias associated with mixed plot characteristics (upland vs flooded) and the different P50 measurement method, we have excluded both ATTO and ZF-2 from the analysis and revised Fig 2 and associated text accordingly.

In the regressions using HSM as the dependent variable and 1–TAC as the predictor (Fig 2 a–c), several plots indeed fall outside the 95% confidence intervals, and the explained variance is moderate (R^2 = 0.29 to 0.57). This pattern indicates that HSM alone does not fully explain the observed variability in TAC across sites.

Then, when we additionally account for long-term hydrological context by modeling 1–TAC as a function of both HSM and cumulative water deficit (CWD) (Figure 2d–f), model performance improves substantially (R^2 = 0.79 to 0.83). In this case, forest plots experiencing different background water availability (as reflected by CWD) align along approximately parallel relationships with distinct intercepts, suggesting that ecosystem resilience captured by TAC reflects not only intrinsic hydraulic resistance but also long-term adaptation to hydrological conditions. We have revised the manuscript accordingly to clarify this interpretation.

line 173: Please define "site-years" here

Response: Thank you. We have removed "site-year" as we only used individual site in the revised manuscript.

line 495-496: Please specify what organs were used for each metric. Were only shoot or branch

Pmin and P50 used? When during the season were measurements taken? If the values were determined earlier in the season you would have positive HSM given a less negative Pmin compared to mid-season.

Response: Thank you for your valuable comment. We have clarified the organs sampled and the timing of measurements in the revised manuscript.

In terms of P50, data was collected from branches. Tree samples were collected from fully sun-exposed, top-canopy branches (or from the highest branches accessible to climbers). P50 values for all sampled species were measured using the pneumatic method (Pereira et al., 2016).

For Pmin, measurements were based on leaf water potential in field condition. Specifically, three to six fully expanded, sun-exposed, top-canopy leaves per individual were sampled at noon (11:00 - 14:30) during the peak of the dry season (specific sample year and month can be found in Extended Data Table 2), ensuring that Pmin reflects maximum seasonal water stress rather than early-season conditions.

Reference:

Pereira, L., Bittencourt, P. R., Oliveira, R. S., Junior, M. B., Barros, F. V., Ribeiro, R. V., & Mazzafera, P. (2016). Plant pneumatics: stem air flow is related to embolism–new perspectives on methods in plant hydraulics. *New Phytologist*, 211(1), 357-370.

line 496-498: Please provide the range of mean annual precipitation and temperature to characterize how widely they differ.

Response: Thank you for your comment. We have added the mean annual precipitation (MAP) and temperature (MAT) ranges in the updated manuscript (**Lines 685–687**): *"Across forest plots, climate conditions spanned a broad gradient, with mean annual precipitation ranging from 1418 mm to 2087 mm, mean annual temperature from 23.46 to 26.63 °C, and cumulative water deficit (CWD) from -1688 to -316 mm/year."* Detailed MAP and MAT values for each plot are provided in Extended Data Table 2.

line 489: For the final estimates of Pmin, was leaf or xylem water potential used? They can be significantly different with enough tissue capacitance.

Response: Thank you for your attention to this detail. The Pmin values used in our analysis represent leaf water potential rather than xylem water potential. This approach is commonly used (Tavares et al., 2023; Barros et al., 2019) as during the dry season stomatal closure reduces leaf transpiration and, consequently the water potential gradient from branch to leave is greatly reduced. We have clarified this definition in the revised manuscript to avoid any ambiguity.

Reference:

Tavares, J. V. *et al.* Basin-wide variation in tree hydraulic safety margins predicts the carbon balance of Amazon forests. *Nature* **617**, 111–117 (2023).

Barros, F. de V. *et al.* Hydraulic traits explain differential responses of Amazonian forests to the 2015 El Niño-induced drought. *New Phytol.* **223**, 1253–1266 (2019).

line 501: Species level traits is not necessarily a bad criterion of hydraulic data. Were the traits not weighted by species composition before your analysis?

Response: We apologize if our methodology was unclear in the original text. Yes, the traits were indeed weighted by species composition. We did not use raw species-level averages; instead, we utilized the basal-area weighted community mean HSM for each plot. This step is essential to explicitly match the community-level hydraulic response to the landscape-scale satellite signal.

Because we relied on the carefully compiled community-level dataset from Tavares et al. (2023), this critical weighting step had already been performed based on extensive forest inventory data. We have revised the Methods section to explicitly detail this scaling approach, making it clear that all HSM values used in our regressions are community-weighted means that represent the specific sampling plot.

line 504: what were the "same methods" used for measuring hydraulic traits? How representative of the population were the species for which you found hydraulic data?

Response: Thank you for your valuable comment. We have revised the manuscript to explicitly clarify that the **pneumatic method** (Pereira et al., 2016) was used for measuring P50 for species in all plots, ensuring methodological consistency. To avoid potential methodological bias and to ensure robustness and comparability across sites, we therefore excluded ATTO and ZF-2 which was included in the original manuscript since P50 measurements at both sites were derived using the bench dehydration approach (Sperry et al., 1988).

Reference:

Pereira, L., Bittencourt, P. R., Oliveira, R. S., Junior, M. B., Barros, F. V., Ribeiro, R. V., & Mazzafera, P. (2016). Plant pneumatics: stem air flow is related to embolism–new perspectives on methods in plant hydraulics. *New Phytologist*, 211(1), 357-370.

Sperry, J., Donnelly, J., Tyree, M. (1988). A method for measuring hydraulic conductivity and embolism in xylem. *Plant, Cell & Environment* 11: 35–40.

To assess representativeness, we report the total number of sample species for each hydraulic trait, total sampled stem basal area, and the relative abundance and dominance of the sampled species (Table 1 below).

At sites FEC, TAM, NVX, and KEN1, the sampled species account for a large proportion of total basal area, indicating that the basal-area weighted mean reliably represents the community.

At five hyperdiverse sites (ALP2, ALP1, SUC, CAX, and MAN), sampled species account for less than 30% of total basal area. However, these forests lack strong dominance by a few species, traits variation is low relative to the mean, and traits are randomly distributed across species dominance distributions. Under such conditions, Barros et al. (2019) shows that species mean values closely approximate community mean trait values. Therefore, even at these sites, available hydraulic data provides a reasonable representation of community-level hydraulic structure.

Table 1. Number of species sampled for each trait per site and the relative dominance of sampled species.

ID	Site	P50	Pmin	HSM	Total sampled basal area in terms of HSM (%)	Sum of the relative abundance of the species which hydraulic traits were measured (in terms of number of stem) (%)	Sum of the relative dominance of the species which hydraulic traits were measured (in terms of basal area) (%)	Reference
1	ALP2	11	10	10	27.32	19.01	16.61	Tavares et al., 2023
2	ALP1	14	14	14	27.16	26.4	23.49	Tavares et al., 2023
3	CAX	18	18	18	25.5	NA	25.50	Tavares et al., 2023, Bittencourt et al 2020
4	FEC	19	15	15	59.43	45.83	27.64	Tavares et al., 2023
5	MAN	17	17	17	13.69	NA	13.69	Tavares et al., 2023, Barros et. al 2019
6	TAM	26	21	21	59.36	56.61	45.3	Tavares et al., 2023
7	NVX	7	7	7	51.38	52.19	60.42	Tavares et al., 2023, Jancoski et al. unpublished
8	SUC	23	23	23	23.90	38.92	24.92	Tavares et al., 2023
9	KEN1	10	11	11	75.16	71.92	45.11	Tavares et al., 2023

Reference:

Tavares, J. V. et al. Basin-wide variation in tree hydraulic safety margins predicts the carbon balance of Amazon forests. *Nature* 617, 111–117 (2023).

Barros, F. de V. et al. Hydraulic traits explain differential responses of Amazonian forests to the 2015 El Niño-induced drought. *New Phytol*. 223, 1253–1266 (2019).

Bittencourt, Paulo RL, et al. "Amazonia trees have limited capacity to acclimate plant hydraulic properties in response to long-term drought." *Global Change Biology* 26.6 (2020): 3569-3584.

line 505: Did these studies measure a representative number of individuals for each species in the plots?

Response: Thank you for the question. Yes, hydraulic traits for each species were measured using plant material collected from at least three individual plants per species within each plot. The reported species-level trait values represent the average across these individuals. We have updated the Extended Data Table 2 to reflect the number of sample species for each plot.

line 569: Were the hydraulic trait measurements taken at the same time? What time during the season were there measured? If P50 was measured on younger tissues your measurements would underestimate the drought tolerance.

Response: Thank you for the comment. The Pmin were sampled during the peak of the dry season and details of sampling year and month of Pmin are listed in the Extended Data Table 2. In terms of the sampling time of the day, they are typically during the noon (11:00 – 14:30).

In terms of the tissue age of P50 measurements, tree samples were collected from fully sun-exposed, top-canopy branches (or from the highest branches accessible to climbers) to ensure accurate measurement of drought tolerance.

Figures:

Figure 3: Suggest remove panel a. It seems unnecessary if no correlations were possible. Additionally, the importance of bimonthly vs quarterly relationships needs to be discussed in the introduction.

Response: Thank you for the suggestion. We have removed panel a in Fig 3.

Figure 2 caption needs better description of the satellites and indicated that those are satellites in the caption.

Response: Thank you for the helpful comment. We have improved the Fig 2 caption to indicate that the TAC values were derived from Landsat time series.

Extended Data figures 1 and 2 do not add to your story. The audience is highly likely to understand how prevalent the research is.

Response: Thank you for the suggestion. We have removed Extended Data figures 1 and 2.

Extended Data Table 1 needs references for the "What does it measure" column

Response: Thank you for the comment. We have added reference for the last column of Extended Data Table 1.

Reviewer #2 (Remarks to the Author):

Review – Song et al. – Nature E&E

The paper by Song et al. makes an explicit link between the widely used resilience indicator temporal autocorrelation and a physiological plant response to water deficits, based on a number of plot locations in the Amazon. I think that the general idea of the paper is sound, but there needs to be some improvement around the methodological treatment of the remote sensing data, as well as in the framing of the paper. The authors claim that they find a general relationship, but this is based on a few stations (covering mostly one month of data each) in a confined geographic location – this is not at all general to global-scale analyses, or to how resilience may or may not be changing through time. The data limitations and caveats need to be made more explicit, as well as the generality of the results. I do not doubt that there is a link between hydrologic sensitivity and temporal autocorrelation, but I am not completely convinced the methods used here are substantial enough for the claims of the authors. I leave some more specific comments below.

Response: Thank you for your time reviewing our manuscript and for your thoughtful and constructive comments. We fully agree with the reviewer that global-scale analyses are critically important for generating broader inferences across regions and vegetation types. However, rigorously validating the physiological basis of a satellite-derived temporal metric (TAC) imposes strict spatiotemporal and methodological constraints that current global hydraulic databases cannot satisfy.

In our study, hydraulic safety margin (HSM) is defined as a location-specific and time-explicit measure of community drought resistance. To link this to satellite-derived TAC, the field data must possess precise geographic coordinates and similar temporal timestamps (e.g., within the same year), and must be measured using a highly standardized methodology to ensure signal-to-noise fidelity. Most existing hydraulic databases, including Smith-Martin et al. (2023), Anderegg et al. (2018), and Knighton et al (2024), do not meet these requirements for direct satellite validation. For example, we evaluated the feasibility of expanding our analysis using Smith-Martin et al. (2023). We found three major barriers for remote sensing validation. (i). Many compiled studies lack exact sampling timestamps (month/year), and frequently aggregate distinct ecological plots under identical, generalized geographic coordinates. This spatial and temporal blurring makes it impossible to extract the correct, corresponding satellite TAC moving window. (ii). The global data compiles P50 values obtained from fundamentally different measurement techniques (bench dehydration, the optical vulnerability technique, and the pneumatic approach). These methods are known to yield systematically different estimates of P50. (iii). When minimum leaf water potential recorded in the field is unavailable, global syntheses often calculate HSM as the difference between leaf turgor loss point and P50, causing HSM definition inconsistency.

These methodological inconsistencies introduce massive artificial variance into HSM estimates that are unrelated to true differences in hydraulic resistance. Attempting a global analysis with this

data would introduce methodological noise that completely obscures the high-sensitivity relationship between true physiological resistance and satellite TAC. Therefore, to isolate the true physiological signal, we restricted our analysis to the Amazon as a necessary, highly controlled natural experiment. The dataset used in this study (Tavares et al., 2023) is globally unique: it provides an extensive community-level sampling (114 tree species, >300 trees) using a single, standardized measurement protocol (the pneumatic approach), with exact spatial GPS and temporal sampling records. While our empirical data is geographically constrained to the Amazon, the Amazon provides an ideal model system. It spans massive hydroclimatic gradients (from extremely wet to seasonally dry) and features immense functional diversity, allowing us to evaluate the resistance–resilience relationship under contrasting environmental constraints. Furthermore, while the specific baseline values of TAC and HSM may vary by biome, the underlying biophysical mechanism we validate here—the fundamental relationship between xylem embolism, loss of canopy conductance, and structural degradation—is governed by universal plant physiological principles. Thus, the finding that TAC successfully captures these hydraulic failure dynamics represents a generalizable validation of the indicator's mechanistic fidelity.

We have explicitly updated the manuscript's Discussion to reflect these data limitations, the strict methodological requirements for satellite validation, and the caveats regarding global generality. We sincerely thank the reviewer for pushing us to clarify these boundaries, which has greatly strengthened the framing of the study.

Reference:

Anderegg, William RL, et al. "Hydraulic diversity of forests regulates ecosystem resilience during drought." *Nature* 561.7724 (2018): 538-541.

Knighton, James, Pablo Sanchez-Martinez, and Leander Anderegg. "A global dataset of tree hydraulic and structural traits imputed from phylogenetic relationships." *Scientific Data* 11.1 (2024): 1336.

Smith-Martin, Chris M., et al. "Hydraulic variability of tropical forests is largely independent of water availability." *Ecology Letters* 26.11 (2023): 1829-1839.

Title – I recommend modifying this to be less general – this is a relationship that was found for a gradient in the Amazon, not a global relationship. I think the work is interesting, but the title (and abstract) should be more explicit about what you actually study: a link between hydrologic safety margins and TAC in the Amazon over a small number of stations.

Response: We thank the reviewer for the comment. The title has been revised to explicitly acknowledge the Amazonian scope of the analysis, and the abstract has been updated accordingly.

Line 65-67 – This explicit link between real recoveries after a disturbance and the theoretical

indicators AC1 and variance was made by Smith et al. 2022, Nature Climate Change (https://www.nature.com/articles/s41558-022-01352-2).

Response: Thank you for raising this point. We have incorporated Smith et al (2022) in the revised manuscript (**Lines 64-67**): "The link between TAC and resilience is supported by theoretical analyses, model simulations[33–35], controlled experiments[15,36,37], and biome-specific empirical studies[5,38-40], and has been further investigated by comparing TAC to observed vegetation recovery following disturbances[41]."

Reference:

[41] Smith, T., Traxl, D. & Boers, N. Empirical evidence for recent global shifts in vegetation resilience. *Nat. Clim. Change* 12, 477–484 (2022).

Line 68-69 – Recent work has made this reliance on data fidelity more explicit: https://arxiv.org/abs/2505.19034

Response: We thank the reviewer for highlighting this recent preprint. We have cited it in the revised manuscript (**Lines 71-74**): "*Atmospheric conditions, sensor-specific limitations, and data-processing choices (e.g. outlier removal and gap-filling[42]), introduce noise that can alter TAC estimates[16,18,42] complicate its ecological interpretation, and drive the contrasting patterns of vegetation resilience trends reported in recent literature[30].*"

Reference:

[42] Liu, T. et al. The influence of data gaps and outliers on resilience indicators. ArXiv Prepr. ArXiv250519034 (2025).

Figure 3 and Lines 310-314 - I think this is a very nice analysis – but would be very specific to the sites chosen. The optimal time sampling and moving windows would be potentially very different for different types of vegetation (e.g., grasslands grow much faster than forests). The specific application here where the sites are all in somewhat similar vegetation types should be sound, but it should be emphasized that these ideal spacings and windows are not universal, and would also vary substantially with e.g. spatial resolution. More plot-scale sites in different vegetation communities would be needed to test how universal this relationship is.

Response: Thank you for the constructive comment. We fully agree that the optimal time sampling and moving windows depend strongly on vegetation types and spatial resolutions.

First, we have revised the manuscript to explicitly emphasize this point. As noted in **Lines 315-324**, we clarified that the identified optimal settings reflect a resonance between the observational frequency and the pace of forest disturbance and recovery, rather than a general rule applicable across ecosystems: *"The choice of VIs, temporal compositing interval, and rolling window of*

Earth observations can drastically influence the reliability of TAC. We found that a bi-monthly compositing interval achieved an optimal resonance, synchronizing the observational frequency with the characteristic timescale of forest perturbation and recovery. However, because functional rhythms and physical structures differ across biomes, these parameter settings cannot be universally applied. For instance, optimal temporal settings must adapt to inherent system dynamics[30], as grasslands exhibit faster growth and shorter turnover rates than forest. Similarly, the choice of VI must account for physical constraints, as high-biomass regions (e.g., dense tropical forests) present well-known saturation challenges for accurate TAC-based resilience estimation[75-77].” Due to the limited availability of field measurements, we are currently unable to evaluate remotely sensed TAC or provide generalized guidance across a broader range of vegetation communities. Expanding this analysis will require additional, standardized field hydraulic data from diverse ecosystems. As such datasets become available, future work will be able to extend across biomes and vegetation types.

Second, we have justified the physiological fidelity of TAC and the rationale for using Amazon forests as the study area (**Lines 339-347**): *“Tracking resilience globally is critical; however, validating highly sensitive satellite metrics requires strict spatiotemporal data. Current global hydraulic syntheses[58,62,78] often lack exact sampling timestamps, aggregate distinct plots, and mix fundamentally different P50 measurement techniques. Because this methodological noise prevents precise satellite validation, we restricted our study to the Amazon—a uniquely standardized dataset[48] serving as a highly controlled natural experiment. Although geographically constrained, the biophysical mechanisms linking xylem embolism to canopy degradation are governed by universal physiological principles, making our validation of TAC’s mechanistic fidelity generalizable.”*

Line 543 – Did you try using single-sensor time series instead? Resilience estimates like TAC can be heavily biased by changes in instrumentation (https://esd.copernicus.org/articles/14/173/2023/, https://www.nature.com/articles/s41558-025-02328-8), so it would be worth making sure that doesn’t bias your TAC too much. Each plot is only over a single year, so you could calculate a TAC for just that year, for example. Alternatively, to keep with your previous framework, you could get the TAC in rolling windows based on individual sensors. You could then feed that forward into your regression, having one TAC value per satellite. As a second note, there is not much discussion of the time period you used – the data from the plots is mostly a single year around 2015 sampled in a single month. The comparison to long-term TAC needs to be better justified, as well as the explicit choices made for the time periods of the satellite data you used.

Response: Thank you for the helpful and thoughtful suggestion. Regarding the use of single vs multi-sensor time series, we chose to use observations from two Landsat sensors at a time to ensure sufficient temporal sampling (Amazon is very cloudy). A single Landsat sensor has a nominal revisit interval of 16 days, and when cloud contamination is considered, particularly in the forest

plots where field hydraulic traits were measured, it becomes difficult to obtain clear observations to support reliable monthly compositing. Using two Landsat sensors substantially improves data availability and temporal continuity. Moreover, since Landsat 5 Thematic Mapper (TM) and Landsat 7 Enhanced Thematic Maper Plus (ETM+) are extremely similar and Landsat 8 and 9's Operational Land Imager (OLI) are almost identical, what we need to adjust is the differences between Landsat 5/7 and Landsat 8/9. To do so, we developed linear regression models (see plots below) for each spectral band and vegetation index used using 10,000 simple random samples (see *Preprocessing of Landsat Time Series* in Methods). The coefficients (slopes and intercepts) were applied to harmonize Landsat 5/7 surface reflectance and vegetation indices to Landsat 8/9-like values. This harmonization step reduces biases arising from differences in sensor characteristics.

<u>Landsat 5/7 vegetation indices VS Landsat 8/9 vegetation indices:</u>

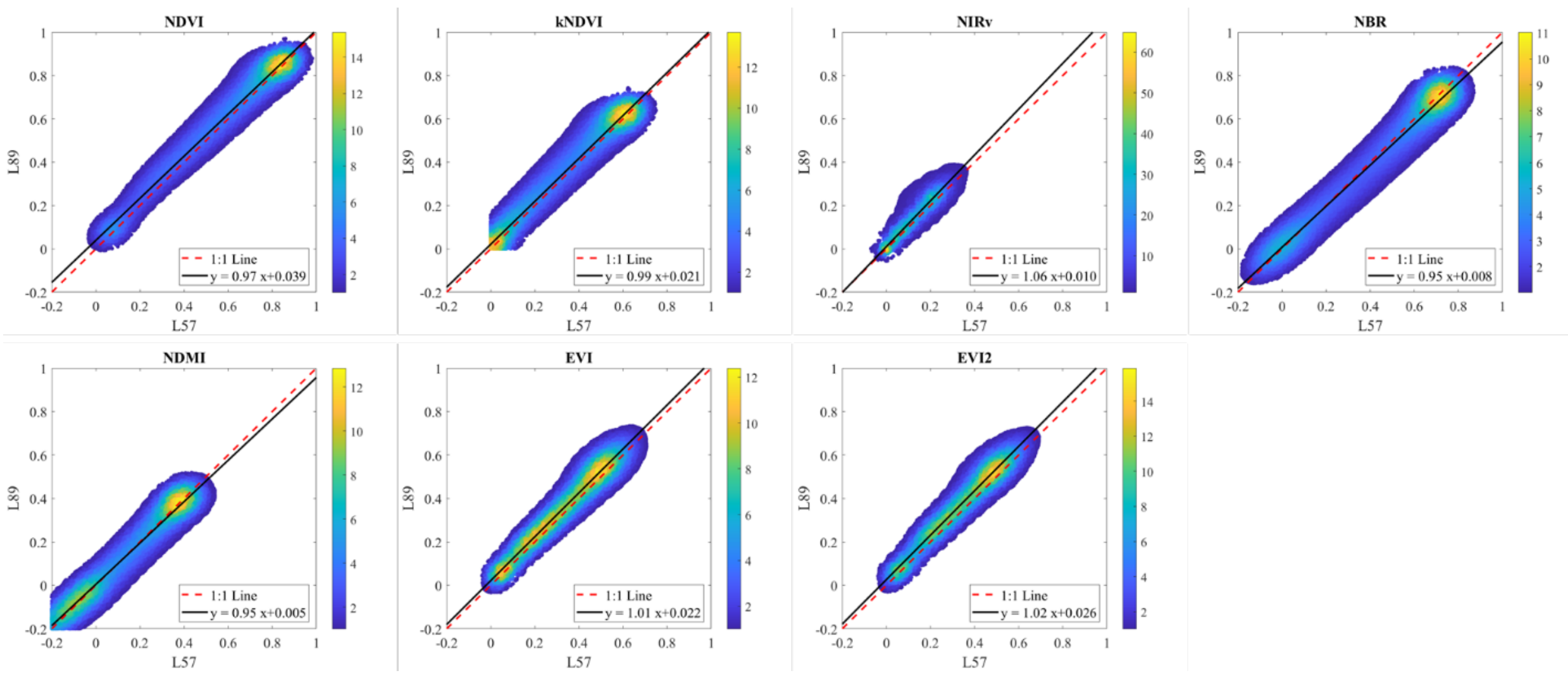

Importantly, to ensure that sensor differences do not bias the inferred relationship between resilience and resistance, we calculated TAC using time series of the individual sensors only, while keeping all other parameters identical to those used in the main text Figure 2 (composite interval = bimonthly, rolling window = 4 years). Then, these TAC estimates were fed into the same regression framework, as suggested. Because single-sensor time series are less dense, the Landsat 7-only analysis yielded 7 valid data points (out of 9 sites). As shown in the panel figure below, the results still demonstrate a **positive relationship between resilience and resistance**, indicating that the association between these two metrics is robust and **not dependent on the use of harmonized multi-sensor observations**. This panel figure is added as Extended Data Fig 5. Applying the same procedure using Landsat 8 alone produced only 3 valid data points due to fewer observations in the rolling window, which is insufficient for fitting reliable linear regression; therefore, we do not interpret this result further. We thank the reviewer for this helpful suggestion, which allowed us to further confirm that the resilience–resistance relationship is robust to potential sensor-specific differences.

Relationships between HSM and TAC derived from Landsat 7-only:

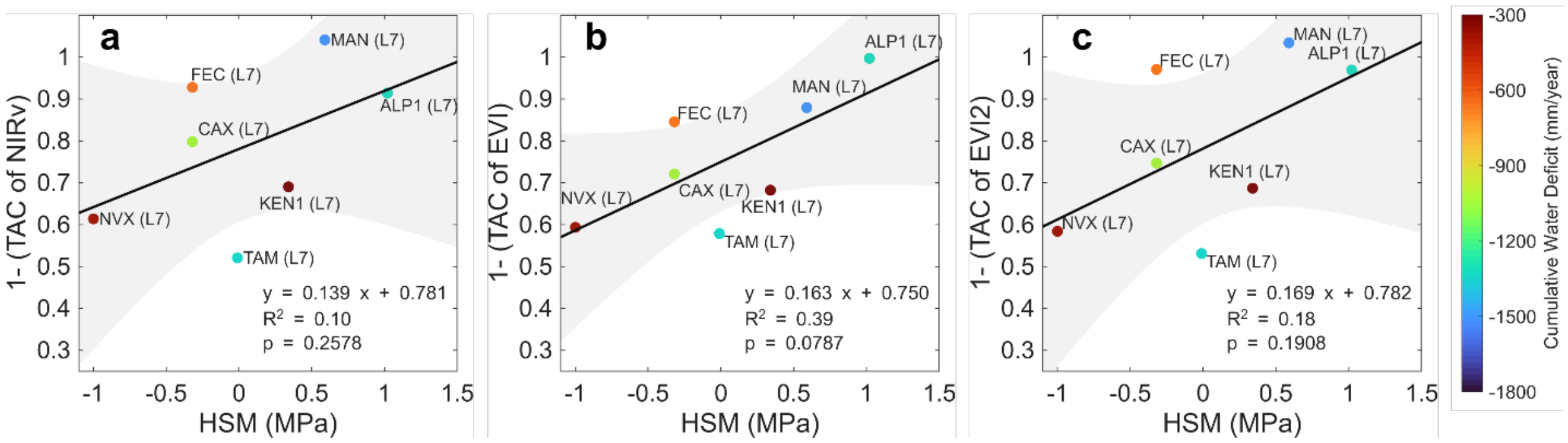

With respect to the second note, we would like to clarify that we did not correlate long-term TAC values with HSM. Instead, we constructed co-timed TAC–HSM pairs by extracting TAC values from the specific time window corresponding to when minimum water potential (Pmin) was measured at each plot (Extended Data Table 2). For a rolling window of *X* years, TAC was calculated using the satellite time series from the *X* years preceding the Pmin measurement. This approach ensures that the analysis captures intrinsic vegetation dynamics while maintaining temporal alignment between the satellite-derived TAC indicator and the in-situ HSM observations. In the updated manuscript, we added the Extended Data Fig 6 to better visualize this rolling window strategy. Thank you again for pointing this out.

Extended Data Fig 6:

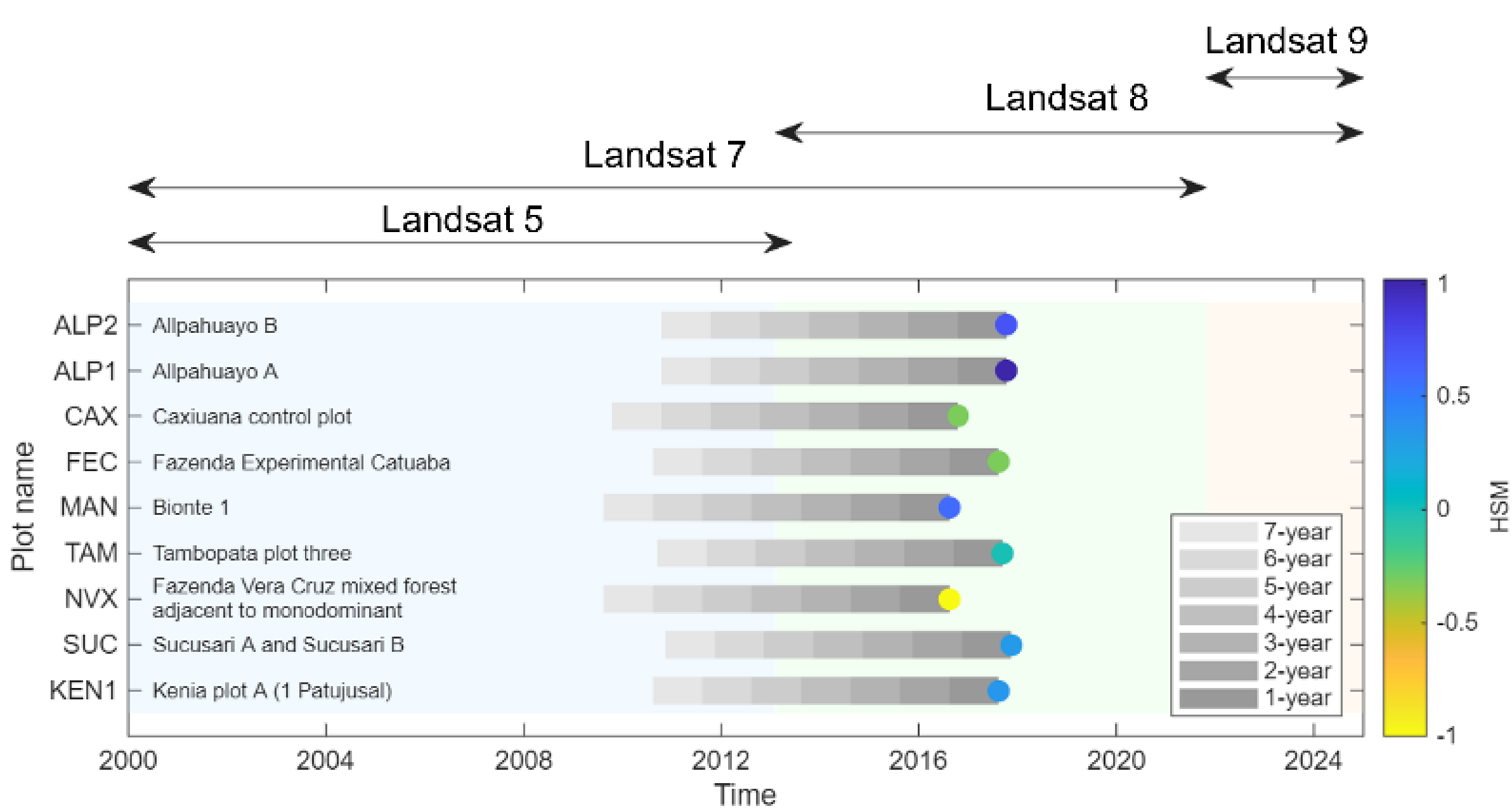

Extended Data Fig 6. Field sample time and corresponding satellite temporal windows used for TAC calculation for each forest plot. Circles indicate the timing of field measurements of Pmin, with colors representing the community-level HSM values. Grey bars denote the rolling temporal windows of satellite observations used to compute TAC preceding each field sampling event, with darker shades indicating shorter windows and lighter shades representing longer windows. Colored background panels illustrate the periods of Landsat sensors included (blue: Landsat 5 and 7; green: Landsat 7 and 8; orange: Landsat 8 and 9), and arrows at the top indicate the operational timelines of these satellite sensors. HSM: hydraulic safety margin, defined as the difference between the minimum stem water potential (Pmin) and P50, the water potential at which 50% of xylem conductivity is lost.

Line 550 – Landsat 7 should be excluded much earlier – there is serious drift in the time of day of data collection: https://tc.copernicus.org/articles/18/5259/2024/, Figure 2.

Response: Thank you for raising this issue. We agree that the orbital drift of Landsat 7 introduces shifts in observation timing in recent years. However, we retained Landsat 7 observations in our analysis without introducing bias for two primary reasons: **First,** our core analysis (HSM vs TAC) predates the severe orbital drift. The field measurements of HSM used to evaluate TAC were collected between 2016 and 2017 (Extended Data Fig 6). Because TAC is calculated using an N-year moving window prior to the field sample date, the satellite time series used for our TAC-HSM regression analysis originates from the years leading up to 2016/2017. Landsat 7's orbital drift only began in 2017, and this mean local overpass time remained close to its historical average until end of 2018 (see Fig 2 from Qiu et al., 2021). Therefore, the satellite observations used to establish the physiological basis of TAC in our study were completely unaffected by the major drift highlighted in your reference. **Second**, our c-factor BRDF normalization effectively mitigates post-2017 drift. For the broader time-series data extending past 2017, we explicitly accounted for the impacts of orbital drift. The primary artifact of orbital drift is a change in the solar zenith angle, which introduces a BRDF effect. To correct this, all Landsat surface reflectance data were processed using c-factor BRDF normalization (Roy et al., 2016). As demonstrated by Qiu et al. (2021), this specific correction greatly removes drift-induced differences, preserving Landsat 7's scientific utility through 2020 (we can continue the analysis to the year of 2021 and the surface reflectance difference after BRDF correction is still extremely small; see Figure (c) below). Furthermore, to avoid the most extreme late-stage drift entirely, we stopped using Landsat 7 data after 2021/10/30 and replaced it with Landsat 9.

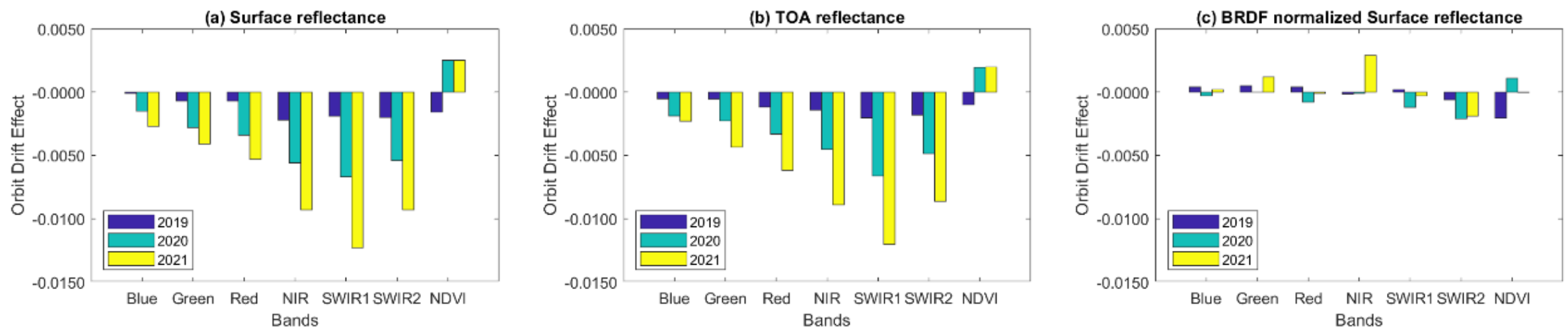

Figure source: https://github.com/GERSL/L7Drift/blob/main/dirft_effects_2021.png

Given the cloud-prone nature of our tropical forest sites, retaining the BRDF-corrected Landsat 7 data provided essential temporal density without compromising data quality. However, we acknowledge that this methodological safeguard should be made explicit to the reader. We have revised the text (**Lines 655-657**) to clarify that the processing steps could reduce potential issues associated with Landsat 7 drift: *"This correction ensured that reflectance values are comparable across dates and seasons*[89] *and reduced the minor effects of Landsat 7 orbital drift*[91]*, providing a stable baseline for downstream analysis."*

Reference:

Roy, D. P., Zhang, H. K., Ju, J., Gomez-Dans, J. L., Lewis, P. E., Schaaf, C. B., ... & Kovalskyy, V. (2016). A general method to normalize Landsat reflectance data to nadir BRDF adjusted reflectance. *Remote Sensing of Environment*, *176*, 255-271.

Qiu, S., Zhu, Z., Shang, R., & Crawford, C. J. (2021). Can Landsat 7 preserve its science capability with a drifting orbit? *Science of Remote Sensing*, *4*, 100026.

Section starting at 568 – Do you fit your harmonic models over all Landsat data for the long period, and then only calculate TAC on the year corresponding to the field data? Or do you use a long-term TAC as a comparison? This is not clear, and could lead to very different results. The irregular and overlapping data sampling is one potential issue of combining sensors, and the comparison of a long-term TAC (from Landsat 5 forward means from the 80s) to a single plot-year is a bit of a reach. The amount of data used for the various different steps in the MS needs to be made much clearer.

Response: Thank you for the question. We fitted the harmonic model over all clear Landsat data for the entire period (Extended Data Fig 3a) using the COntinuous monitoring of Land Disturbance (COLD) algorithm, obtained residual term from the vegetation index time series after detrending and de-seasonality (Extended Data Fig 3b), and then calculated rolling-window TACs from 2000 onwards (Extended Data Fig 3c). See **Lines 699 – 732** in the revised manuscript for more details. To compare with the field data, we used TAC value from the corresponding year and month. We

did not use the long-term average TAC because resilience is a time-varying metric and should be evaluated with co-timed field measurements.

In terms of combining data from multiple sensors, we chose to use data from 2000 to make sure two Landsat-sensors were available throughout the study period, e.g. 2000-2013: Landsat 5 and 7. 2013-2021: Landsat 7 and 8. 2021-2024: Landsat 8 and 9.

Reviewer #2 (Remarks on code availability):

I was not able to find any of the python scripts in the Github (they are listed in the code repository, but are not available on the Github), so cannot comment on the Earth Engine processing chain. I am not proficient in Matlab so cannot comment on the application of the main codes of the MS.

Response: We thank the reviewer for carefully examining the code availability and for pointing out these issues. We have updated the GitHub repository accordingly to address the reviewer's concerns.

1. All Python scripts used for downloading Landsat data via the Google Earth Engine API and for performing the c-factor BRDF harmonization have now been added to the repository.

2. We have expanded the methodological description in the README.md to clearly explain the logic of the core analysis implemented in MATLAB, ensuring transparency and accessibility independent of programming language.

3. In addition, we have added detailed code comments, example scripts, and usage instructions to the repository to improve accessibility and facilitate reproducibility.

Reviewer #3 (Remarks to the Author):

Overall, the manuscript presents a valuable and timely contribution to understanding forest resilience through remote sensing. However, several aspects require attention to strengthen its scientific rigor and contextual accuracy:

Response: Thank you very much for your positive evaluation of our work. We have carefully revised the manuscript based on your comments.

1. Involvement of Original Data Contributors

The study relies heavily on Brazilian field measurements and previously published hydraulic trait datasets. Authors of these original studies should be involved as co-authors or acknowledged collaborators to ensure proper attribution, compliance with local research permits, and accurate ecological interpretation.

Response: Thank you for the comment. We fully agree that proper attribution and collaboration are essential when building upon field-based hydraulic datasets. To ensure appropriate involvement, we contacted the first authors of the primary studies on which our analysis relies (Tavares et al., 2023; Barros et al., 2019; Bittencourt et al., 2020; and Jancoski et al.) and invited them to review the manuscript and consider co-authorship. Two have joined as co-authors, and one preferred to be acknowledged. Their input has helped ensure accurate ecological interpretation and appropriate representation of the original field measurements.

We also revised the manuscript to more explicitly cite these foundational studies throughout the main text and Methods section to ensure clear attribution (**Lines 101-103**): *"Because quantifying community-level HSM[49,55,56] requires substantial field investment (see Methods), such data have only recently become available, providing a rare opportunity to examine the mechanisms underlying satellite-derived TAC."* and (**Lines 122-125**): *"…our study utilizes two key datasets: a collection of plot-specific, time-explicit HSM measurements from 9 Amazonian forest plots (obtained from Ref[49] which also used data from Ref[55,56]; see details in Extended Data Fig 1 and Extended Data Table 2) and the 30 m spatial resolution of Landsat data."*

In addition, we revised the Extended Data Table 2 with referencing the material used in the compilation to further enhance the acknowledgement of these field studies.

The hydraulic traits used in this study were obtained from a ForestPlots.net data package. This dataset is licensed under a Creative Commons Attribution-ShareAlike 4.0 International License (CC-BY-SA 4) and our study uses this publicly available dataset in accordance with their stated terms of use. In terms of local research permits, except for the NVX site which only requires owner's permit since it's a private area, sample collections in the rest forest plots were granted with appropriate national research authorizations and institutional oversight, as documented in the Reporting Summary of Tavares et al., 2023.

Reference:

Tavares, J. V. *et al.* Basin-wide variation in tree hydraulic safety margins predicts the carbon balance of Amazon forests. *Nature* **617**, 111–117 (2023).

Barros, F. de V. *et al.* Hydraulic traits explain differential responses of Amazonian forests to the 2015 El Niño-induced drought. *New Phytol.* **223**, 1253–1266 (2019).

Bittencourt, Paulo RL, et al. "Amazonia trees have limited capacity to acclimate plant hydraulic properties in response to long-term drought." *Global Change Biology* 26.6 (2020): 3569-3584.

Jancoski et al. Unpublished.

2. Contextualizing Novelty Claims

While the insights on TAC and its physiological link are important, claims of being the "first mechanistic investigation" should be moderated. TAC has been mechanistically explored in other domains (e.g., aquatic ecosystems, microbial systems, neuroscience, climate modeling). The true novelty here lies in the field-based physiological validation for forests using hydraulic traits and the methodological guidance for remote sensing applications.

Response: We thank the reviewer for this helpful comment. We agree that the novelty of this research lies in the unique combination of field-based physiological investigation of satellite time series-based forest resilience and methodological guidance. Thank you for highlighting this! We have removed "the first mechanistic investigation" to moderate our claims (**Lines 269-271**): *"This study provides a mechanistic explanation for a critical question in ecosystem science: whether satellite-derived lag-1 temporal autocorrelation (TAC) reflects ecologically meaningful resilience dynamics."*

3. Strength of Remote Sensing Insights

The discussion on observation frequency, rolling window size, and vegetation index selection is a major strength of the paper. These methodological clarifications are highly valuable for improving TAC-based resilience monitoring.

Response: Many thanks for this positive feedback.

4. Compositing and Gap-Filling Methods

More transparency is needed regarding the compositing approach and gap-filling procedures described in Extended Data Fig. 6. Gap-filling can introduce bias in TAC estimates, and its impact should be explicitly evaluated. Ideally, the authors should either avoid gap-filling or quantify its effect on TAC calculations to ensure robustness.

Response: Thank you very much for this valuable comment. We agree that the potential impact of gap-filling on TAC calculations should be evaluated. To address this concern, we conducted additional analysis comparing long-term TAC values derived from non–gap-filled and gap-filled time series. Specifically, we tested thresholds allowing up to one (conseGap = 1) and two (conseGap = 2) consecutive missing observations using a set of randomly collected forest samples (n = 2725).

As shown in the figure below, permitting one or two consecutive missing observations slightly increased long-term TAC estimates relative to the no-gap-filling baseline, suggesting modest inflation. However, gap-filling substantially improved data availability by increasing the proportion of valid pixels retained for analysis. These results indicate a trade-off between minimizing interpolation-induced bias and maximizing spatial coverage. **Therefore, we selected conseGap=1 to balance TAC calculation bias and the number of valid data points.**

In addition, we explicitly evaluated whether gap-filling influenced the observed TAC–HSM relationship. For both bimonthly and quarterly composite intervals, the strength and direction of the TAC–HSM association remained consistent across gap-filling thresholds, suggesting that our main conclusions are robust to interpolation choices.

<u>Trade-off between interpolation-induced bias and data availability</u>:

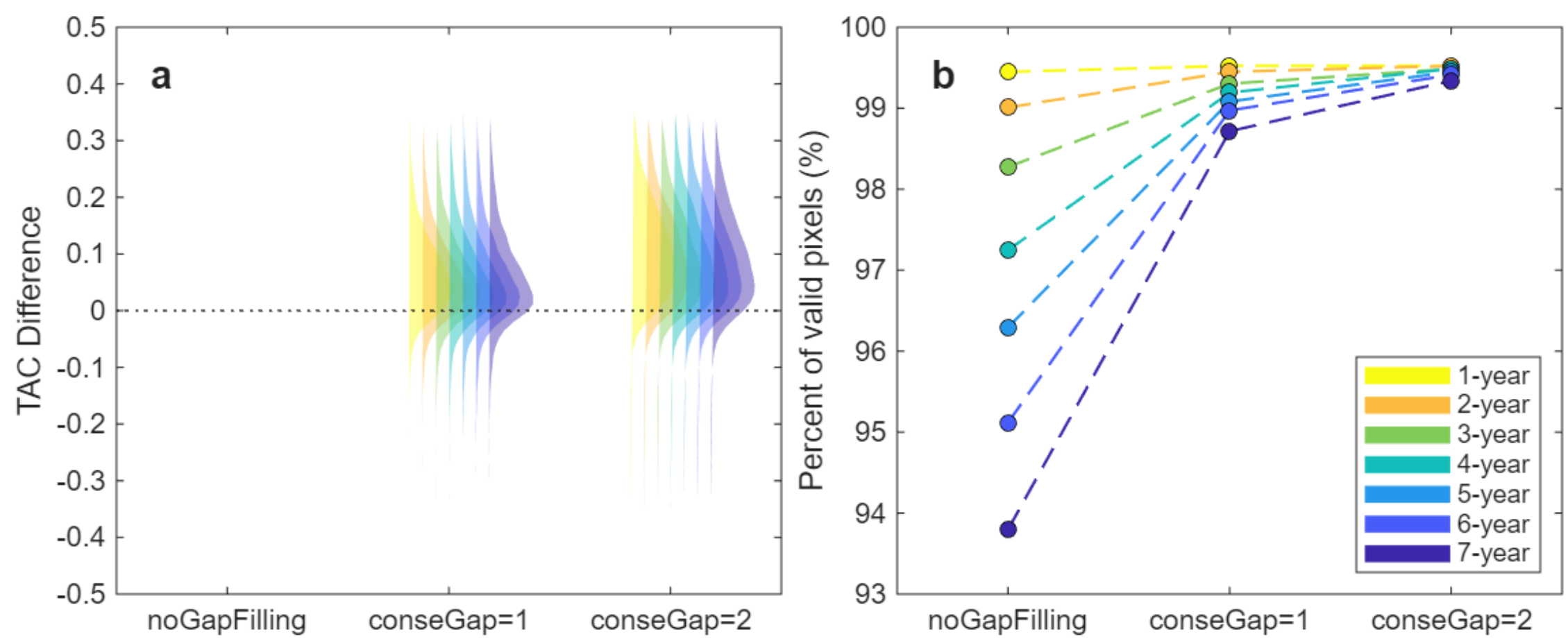

<u>TAC-HSM relationship quantified by R^2 (bimonthly):</u>

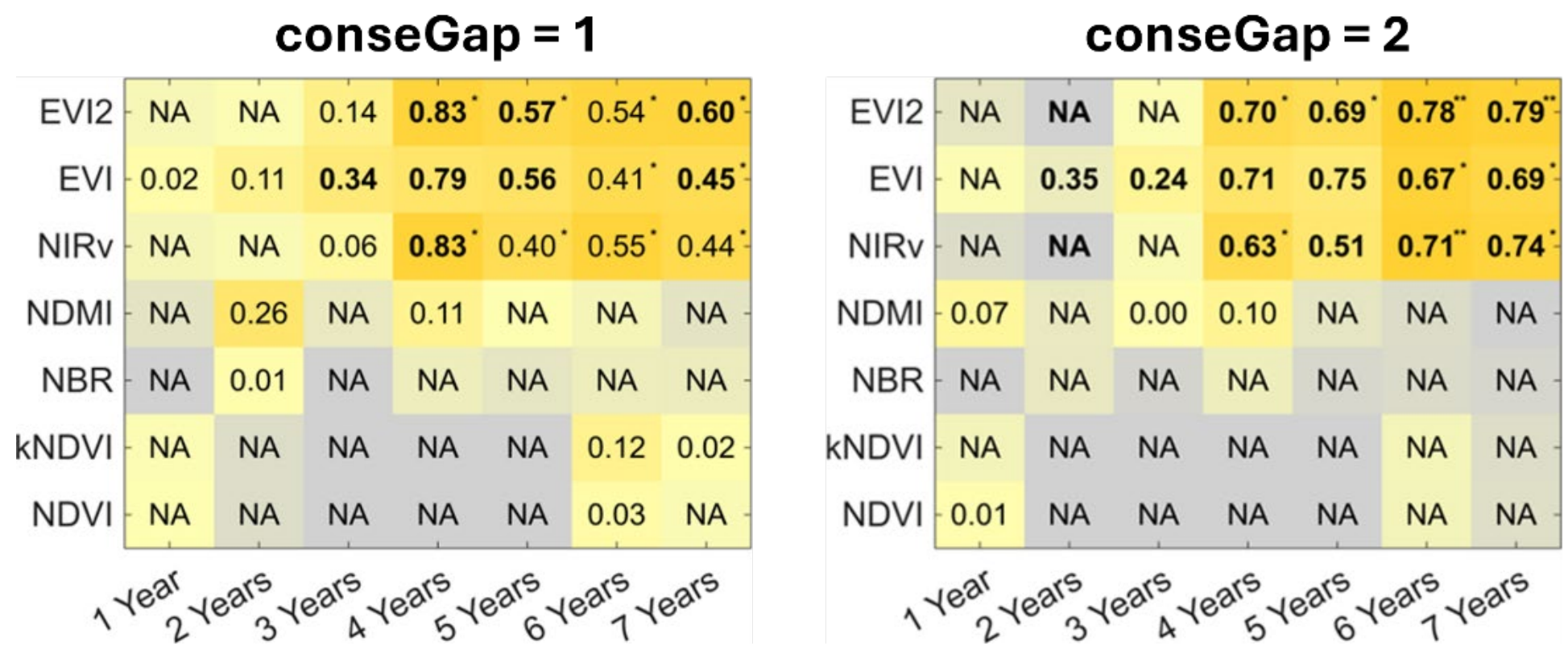

TAC-HSM relationship quantified by R^2 (quarterly):

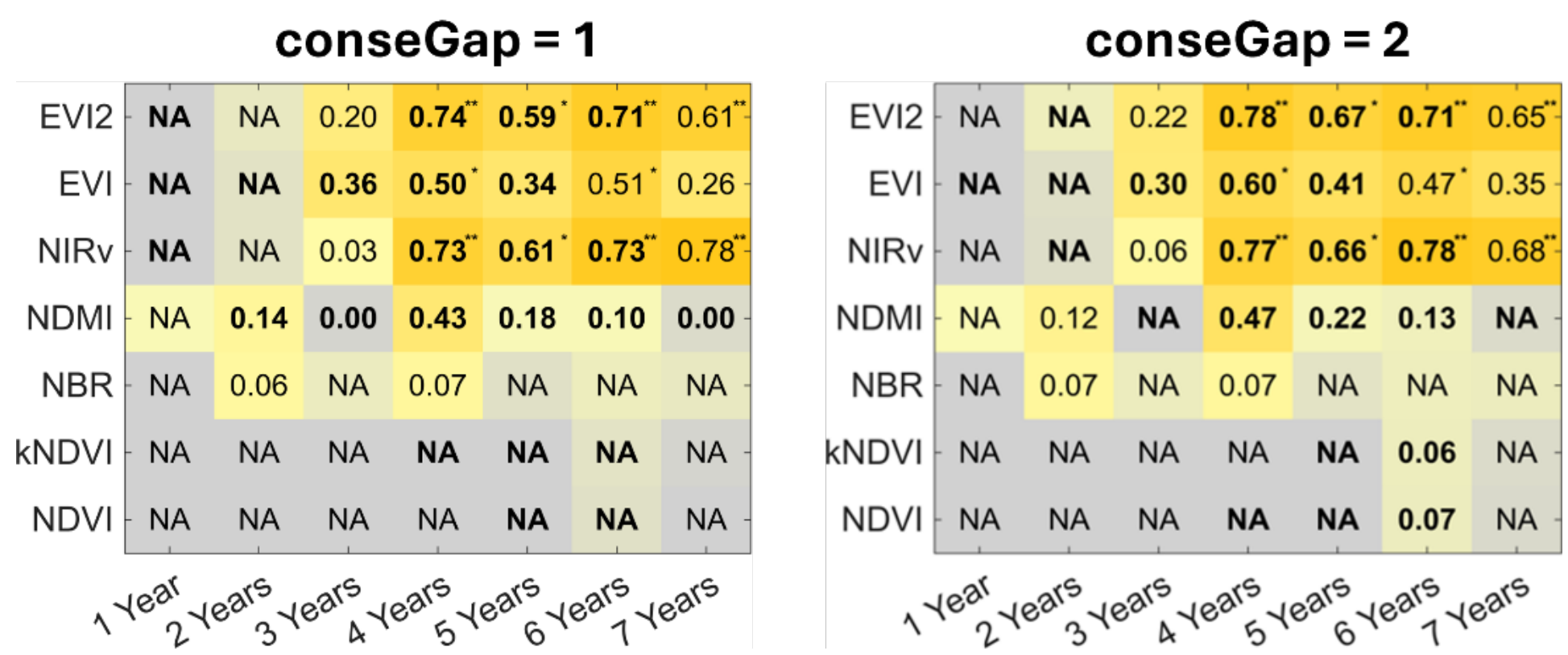

(1 – TAC of EVI2) vs HSM at bimonthly, 5-year rolling window with respect to no-gap-filling and different gap-filling thresholds, note that conseGap=0 (left panel) has 8 points for analysis due to the lack of TAC at the forest plot SUC:

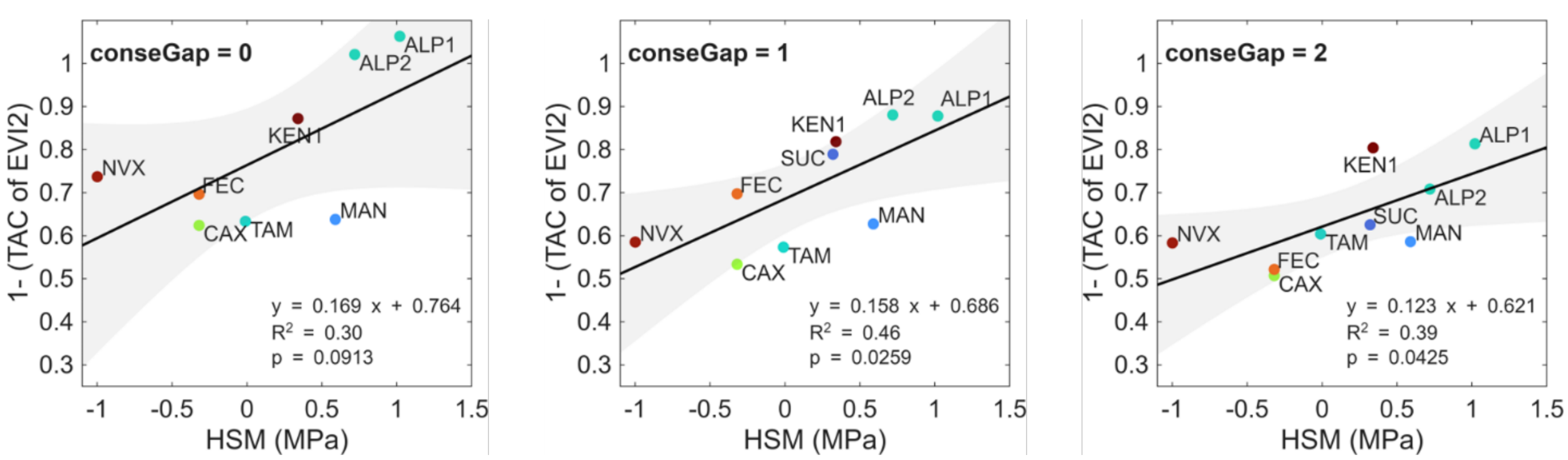

In more detail explained:

1. Given that the validation relies on Brazilian insitu hydraulic trait datasets collected under local permits, the paper would benefit from the inclusion of Brazilian collaborators (e.g., investigators from INPA/UNICAMP or original dataset authors) as coauthors or acknowledged contributors.

This will help ensure accurate ecological interpretation of plot contexts (upland vs. floodplain) and transparent reporting of permits (SISBIO) and data licenses (e.g., CC BYSA via ForestPlots).

Response: Thank you for this suggestion. As noted in our response to the previous comment, we have taken steps to ensure appropriate involvement of investigators associated with the original hydraulic trait datasets and clarified the permits and data licenses. We contacted several key contributors of the primary studies (Tavares et al., 2023; Barros et al., 2019; Bittencourt et al., 2020; and Jancoski et al.), invited them to review the manuscript and consider co-authorship, and incorporated their input to strengthen the ecological interpretation of plot contexts (e.g., all plots used in the analysis are upland forests).

2. The claim that this study provides the first mechanistic investigation of TAC should be qualified. Temporal autocorrelation has been mechanistically linked to resilience in multiple domains beyond terrestrial forests. For example, Shinn et al. (Nature Neuroscience, 2023) demonstrated that TAC explains functional brain network topology and state transitions, highlighting its role in recovery dynamics in neural systems. Similarly, Lenton et al. (Nature Communications, 2023) applied TAC as an early-warning metric for climate tipping points in Earth system models, and Batt et al. (PLOS ONE, 2019) used TAC to detect resilience loss in lake ecosystems approaching eutrophication. These studies show that TAC's mechanistic interpretation as an indicator of slowing recovery is well established across disciplines; the novelty here lies in the field-based physiological validation for forests using hydraulic traits, not in being the first mechanistic study overall.

Suggested references to include:
Shinn M. et al. Functional brain networks reflect spatial and temporal autocorrelation. Nature Neuroscience 26, 867–878 (2023).
Lenton T. M. et al. Early warning of climate tipping points from autocorrelation signals. Nature Communications 14, 498 (2023).
Batt R. D. et al. Detecting critical slowing down in lakes approaching eutrophication. PLOS ONE 14, e0216362 (2019).

Response: Thank you for the valuable comment and for highlighting the novelty of our study. We have revised the manuscript to make the narrative more specific to forest resilience and to better clarify the unique contributions of this work. In addition, we have incorporated the suggested references to better position our study within existing literature. The revised text (**Lines 61-69**) reads: *"While satellite TAC has been rapidly adopted to detect global resilience trends[5,17], identify the drivers of resilience loss[4,31], and forecast critical transitions and tipping points[8,32], this uptake has outpaced the evaluation of its underlying physiological mechanisms. The link between TAC and resilience is supported by theoretical analyses, model simulations[33–35], controlled experiments[15,36,37], and biome-specific empirical studies[5,38-40], and has been further investigated by comparing TAC to observed vegetation recovery following disturbances[41]. Yet, large-scale forest resilience assessments rely almost exclusively on TAC derived from satellite time series,*

often lacking the field-based physiological validation required to confirm these signals." Thank you again for your thorough feedback.

Reference:

[34] Lenton, T. M. Early warning of climate tipping points. Nat. Clim. Change 1, 201–209 (2011).

[35] Shinn, M. et al. Functional brain networks reflect spatial and temporal autocorrelation. Nat. Neurosci. 26, 867–878 (2023).

[37] Batt, R. D., Eason, T. & Garmestani, A. Time scale of resilience loss: implications for managing critical transitions in water quality. PLoS One 14, e0223366 (2019).

Overview of existing studies and results already published to clarify the novelty of the current manuscript. The following studies should be properly cited and included in the current manuscript:

Mechanistic Underpinnings of TAC
• Physiological Link: TAC is mechanistically linked to plant hydraulic safety margin (HSM_P50), a key trait for drought resistance. Field data from tropical forests show a strong correlation between HSM_P50 and the resilience proxy (1-TAC), indicating that community-level drought resistance constrains forest recovery speed and resilience (Tavares et al., 2023; Oliveira et al., 2021).

Response: Thank you. We have cited the suggested studies and emphasized the physiological link between TAC and HSM (**Lines 274-276**): *"...we establish that the most resilient forests—those recovering fastest from non-catastrophic perturbations—are those physiologically best equipped to resist stress[49,53]."*

Reference:

[49] Tavares, J. V. et al. Basin-wide variation in tree hydraulic safety margins predicts the carbon balance of Amazon forests. Nature 617, 111–117 (2023).

[53] Oliveira, R. S. et al. Linking plant hydraulics and the fast–slow continuum to understand resilience to drought in tropical ecosystems. New Phytol. 230, 904–923 (2021).

• Critical Slowing Down: Theoretical and empirical studies confirm that as ecosystems approach critical thresholds (e.g., due to drought), TAC increases—a phenomenon known as "critical slowing down"—signaling reduced resilience and higher risk of abrupt transitions (Smith et al., 2022; Forzieri et al., 2022; Smith & Boers, 2023; Zhang et al., 2025; Wu et al., 2024).

Response: Thank you for the suggestion. We have incorporated these references in the revised manuscript (**Lines 52-54**): *"Among these, lag-1 temporal autocorrelation (TAC) has been widely*

adopted as a proxy for ecosystem resilience[4,5,8–10,17–22]*."* (**Lines 64-67**): *"The link between TAC and resilience is supported by theoretical analyses, model simulations*[33–35]*, controlled experiments*[15,36,37]*, and biome-specific empirical studies*[5,38-40]*, and has been further investigated by comparing TAC to observed vegetation recovery following disturbances*[41]*."*

Reference:

[5] Forzieri, G., Dakos, V., McDowell, N. G., Ramdane, A. & Cescatti, A. Emerging signals of declining forest resilience under climate change. *Nature* 608, 534–539 (2022).

[9] Wu, X., Xu, H., Pang, J. & Zhang, Z. Declining resilience of forest carbon sinks linked to increased atmospheric water deficit during droughts in the Northern Hemisphere. *Environ. Res. Lett.* 20, 014055 (2024).

[22] Zhang, L. et al. Remote Sensing and Critical Slowing Down Modeling Reveal Vegetation Resilience in the Three Gorges Reservoir Area, China. *Remote Sens*. 17, 2297 (2025).

[40] Smith, T. & Boers, N. Global vegetation resilience linked to water availability and variability. *Nat. Commun*. 14, 498 (2023).

[41] Smith, T., Traxl, D. & Boers, N. Empirical evidence for recent global shifts in vegetation resilience. *Nat. Clim. Change* 12, 477–484 (2022).

Methodological Reliability and Limitations

• Influence of Water Availability: TAC-based resilience is strongly modulated by background water availability and cumulative water deficit, with models incorporating both explaining up to 62% of resilience variance (Smith & Boers, 2023; Tavares et al., 2023; Peters et al., 2021).

Response: Suggested reference cited. The updated text (**Lines 169-170**) reads as: *"The inclusion of CWD markedly improved the model fitness, with the full model explaining up to 83% of the variance in resilience.*[40,49,68]*"*

Reference:

[40] Smith, T. & Boers, N. Global vegetation resilience linked to water availability and variability. *Nat. Commun*. 14, 498 (2023).

[49] Tavares, J. V. et al. Basin-wide variation in tree hydraulic safety margins predicts the carbon balance of Amazon forests. *Nature* 617, 111–117 (2023).

[68] Peters, J. M. et al. Living on the edge: A continental-scale assessment of forest vulnerability to drought. *Glob. Change Biol.* 27, 3620–3641 (2021).

• Vegetation Index and Temporal Resolution: The reliability of TAC as a resilience indicator depends on the choice of vegetation index, temporal frequency of satellite observations, and rolling window size. High-biomass regions (e.g., dense tropical forests) present challenges for accurate TAC-based resilience estimation (Smith & Boers, 2023; Müller et al., 2024; Smith et al., 2023).

Response: Suggested reference cited. The updated text (**Lines 315-324**) reads as: *"The choice of VIs, temporal compositing interval, and rolling window of Earth observations can drastically influence the reliability of TAC. ... Similarly, the choice of VI must account for physical constraints, high-biomass regions (e.g., dense tropical forests) present well-known saturation challenges for accurate TAC-based resilience estimation[75-77]."*

Reference:

[75] Müller, M., Olsson, P.-O., Eklundh, L., Jamali, S. & Ardö, J. Response and resilience to drought in northern forests revealed by Sentinel-2. *Int. J. Remote Sens*. 45, 5130–5157 (2024).

[76] Smith, T. & Boers, N. Reliability of vegetation resilience estimates depends on biomass density. *Nat. Ecol. Evol*. 7, 1799–1808 (2023).

[77] Smith, T. et al. Reliability of resilience estimation based on multi-instrument time series. *Earth Syst. Dyn*. 14, 173–183 (2023).

• Fragmentation and Biome Differences: The relationship between TAC and resilience varies by biome and is influenced by landscape fragmentation, with different patterns observed in tropical, temperate, and boreal forests (Su et al., 2025; Forzieri et al., 2022; Lv et al., 2025).

Response: We appreciate your suggestion and included this to the updated manuscript (**Lines 64-67**): *"The link between TAC and resilience is supported by theoretical analyses, model simulations[33–35], controlled experiments[15,36,37], and biome-specific empirical studies[5,38–40], and has been further investigated by comparing TAC to observed vegetation recovery following disturbances[41]."*

Reference:

[5] Forzieri, G., Dakos, V., McDowell, N. G., Ramdane, A. & Cescatti, A. Emerging signals of declining forest resilience under climate change. *Nature* 608, 534–539 (2022).

[38] Lv, X., Chen, G., Wang, Q. & Chen, T. Exploring the resilience of global vegetation ecosystem: Nonlinearity, driving forces, and management. *J. Environ. Manage*. 377, 124634 (2025).

[39] Su, Y. et al. Pervasive but biome-dependent relationship between fragmentation and resilience in forests. *Nat. Ecol. Evol*. 9, 1670–1684 (2025).

References

Su, Y. et al. (2025). Pervasive but biome-dependent relationship between fragmentation and resilience in forests. Nat. Ecol. Evol.
Smith, T., Traxl, D., & Boers, N. (2022). Empirical evidence for recent global shifts in vegetation resilience. Nat. Clim. Change, 12, 477–484.
Forzieri, G. et al. (2022). Emerging signals of declining forest resilience under climate change. Nature, 608, 534–539.
Smith, T., & Boers, N. (2023). Global vegetation resilience linked to water availability and variability. Nat. Commun., 14.
Smith, T., & Boers, N. (2023). Reliability of vegetation resilience estimates depends on biomass density. Nat. Ecol. Evol., 7, 1799–1808.
Müller, M. et al. (2024). Response and resilience to drought in northern forests revealed by Sentinel-2. Int. J. Remote Sens., 45, 5130–5157.
Tavares, J. et al. (2023). Basin-wide variation in tree hydraulic safety margins predicts the carbon balance of Amazon forests. Nature, 617, 111–117.
Zhang, L. et al. (2025). Remote sensing and critical slowing down modeling reveal vegetation resilience in the Three Gorges Reservoir Area, China. Remote Sens.
Wu, X. et al. (2024). Declining resilience of forest carbon sinks linked to increased atmospheric water deficit during droughts in the Northern Hemisphere. Environ. Res. Lett., 20.
Lv, X. et al. (2025). Exploring the resilience of global vegetation ecosystem: Nonlinearity, driving forces, and management. J. Environ. Manag., 377, 124634.
Oliveira, R. et al. (2021). Linking plant hydraulics and the fast-slow continuum to understand resilience to drought in tropical ecosystems. New Phytol.
Peters, J. et al. (2021). Living on the edge: A continental-scale assessment of forest vulnerability to drought. Glob. Change Biol., 27.
Smith, T. et al. (2023). Reliability of resilience estimation based on multi-instrument time series. Earth Syst. Dyn.

Response: We appreciate your valuable comment. In the revised manuscript, we have incorporated the suggested citations to more accurately position our work within the existing publications.

Reviewer #3 (Remarks on code availability):

The public release of the TAC-HSM code is a strong step toward transparency and reproducibility. The repository appears to contain MATLAB/Python scripts for TAC computation, Landsat harmonization, RF models to isolate climate autocorrelation, and plotting/analysis workflows (folders such as TAC, L57_L89_Harmonization, RFmodel, and scripts like runTAC_Landsat_allSample.m, obtainEnhancedTAC_FieldSample.m). However, based on the GitHub page, I cannot assess execution quality, test coverage, or long-term maintenance. The repo shows only a handful of commits and no formal releases or recent updates, suggesting it is currently static. [github.com], [github.com]

Actionable suggestions (minimal burden, high impact):

Add a Reproducibility README with a one-command run

Response: Thank you. We have revised README substantially. Because the workflow involves both Python and MATLAB components that require environment-specific configuration, a strict one-command execution is not currently feasible. Instead, we have added a clearly documented run_pipeline.txt file that outlines the full sequential pipeline in a structured, step-by-step format.

Include exact software versions (MATLAB 2023b, Python 3.10), package lists, OS details, and a small sample dataset so that users can run an end-to-end test in minutes. Provide expected outputs (e.g., test TAC values, R^2) for verification.

Response: Thank you. We have included the suggested details to improve reproducibility.

Environment files & containers

Publish a requirements.txt/environment.yml for Python, and (if feasible) a Dockerfile or conda environment to standardize dependencies. A simple container greatly increases reproducibility for long-lived projects.

Response: Thank you. We have added environmental.yml and create_env.bash for the two python-based folders, /GEE-LandsatTimeSeries (for downloading pixel-level Landsat time series from Google Earth Engine) and /c_factor_brdf_python (for conducting bidirectional reflectance distribution function harmonization on the surface reflectance) in the repository.

Versioning & releases

Tag a v1.0 GitHub Release with notes describing what's included, what's tested, and the figures/results reproduced. Add semantic versioning for future updates. Currently, there are no releases.

Response: Thank you for the comment. We have released the v1.0 GitHub.

Basic tests

Provide small unit/regression tests (e.g., TAC on synthetic signals; harmonization checks; RF model determinism with fixed seeds). Even a minimal CI (GitHub Actions) that runs these tests signals ongoing maintenance.

Response: Thank you for valuable suggestions. We have included the /Tests folder in the Github to support lightweight TAC and regression tests.

Data availability pointers

If large inputs cannot be tracked, add README_data.md with links and checksums (e.g., ForestPlots/Tavares tables, ERA5-Land pointers) and a script to fetch/prepare small subsets to validate the pipeline.

Response: All satellite and field data used in the analysis are included in the Github repository under the /Data folder. For the climate data extracted from ERA5-Land, we have included a google drive link for access. For the forest cover maps obtained from the Global 2010 Tree Cover (30 m), a google drive link storing those maps as well as a matlab script for downloading them are added.

Repository housekeeping

Replace .asv autosave files with stable .m scripts to avoid confusion (runTAC_Landsat_allSample.asv, etc.).

Response: Thank you for the comment. We've removed all .asv files.

Add a license (e.g., MIT) and a CONTRIBUTING.md to clarify reuse and maintenance plans. Response: Thank you. We have added a MIT license and a CONTRIBUTING.md file to the GitHub repository.

Response to reviewers

We appreciate reviewers for their helpful feedback. The specific comments (in black) are responded to point by point below (in **blue**), and the revisions are indicated in ***green***. Below are the revisions implemented in the updated manuscript:

Reviewer #1 (Remarks to the Author):

Thank you for your thorough responses to my comments. Your work is exciting and novel--merging plant physiology and remote sensing.

Response: Thank you for your positive evaluation of our work!

I have a few minor comments:

line 81: Missing an "s" in assess

Response: Thank you. Typo fixed.

line 250: Suggest to replace "calculated" with "represented"

Response: Revised. Thank you for the suggestion.

line 252-253: Suggest to replace "integrates embolism resistance to actual plant operation" to ""

Response: Thanks. The have revised the sentence (**Lines 85-86**) to *"The hydraulic safety margin (HSM[51]) is defined as the distance from the plant water potential during the peak of the dry season (Pmin) to P50."*

line 260-61: Suggest to replace "season decreases in water availability"

Response: Thank you. The text (**Lines 92-93**) has been revised to read: *"drought refers to seasonal decreases in water availability that are within the bounds of the local climate…"*

Reviewer #1 (Remarks on code availability):

For clarity to future readers it may be useful to remove folders and files that not involved in the analysis. I appreciate the .txt instructions but there are many files that are not sourced.

Response: Thank you for this helpful suggestion. We have streamlined the repository by removing unnecessary files and folders and reorganizing the remaining scripts and functions. We have also updated the documentation to clearly indicate the workflow and the role of each script.

Reviewer #2 (Remarks to the Author):

Review – Song et al. – Nature E&E – Revision 1

I would like to thank the authors for their thorough Revision – the majority of my original comments were addressed. I have two remaining questions about the ED figures:

ED3 – Negative TAC? That implies high is always followed by low, so there is no recovery process. How do you interpret that in your framework? In other work negative TAC is masked as data noise.

Response: We thank the reviewer for raising this excellent point. The interpretation of negative TAC values remains an ongoing discussion in the literature. A negative TAC can theoretically reflect rapid negative feedback, meaning the ecosystem recovers faster than the observation interval, exhibiting even greater resilience than TAC = 0 (Forzieri et al., 2022; Zhang et al., 2024; Wang et al., 2024). Conversely, others argue that values below zero may simply represent high-frequency environmental or sensor noise (Smith and Boers, 2023; Liu et al., 2026).

In our study, we chose to retain the raw negative values. By using (1 - TAC) as our resilience proxy, a negative TAC correctly places these sites at the highest end of the resilience spectrum (yielding a proxy value > 1), consistent with approaches in recent resilience literature. However, we fully admit that distinguishing whether a negative TAC represents true biological overcompensation or simply noise is difficult and requires further research. We have added a brief note to the manuscript to clarify our approach and acknowledge this uncertainty (Lines 884-888): *"In this framework, negative TAC values were retained, placing them at the highest end of the resilience spectrum (1-TAC > 1). We acknowledge that while negative TAC may reflect rapid biological recovery, it can also be influenced by high-frequency noise[42,76]; determining the exact ecological significance of TAC < 0 remains an important area for future research."*

Reference:

Forzieri, G., Dakos, V., McDowell, N. G., Ramdane, A., & Cescatti, A. (2022). Emerging signals of declining forest resilience under climate change. *Nature*, 608(7923), 534-539.

Zhang, Y., Wang, J. A., Berner, L. T., Goetz, S. J., Zhao, K., & Liu, Y. (2024). Warming and disturbances affect Arctic-boreal vegetation resilience across northwestern North America. *Nature Ecology & Evolution*, 8(12), 2265-2276.

Wang, H., Ciais, P., Sitch, S., Green, J. K., Tao, S., Fu, Z., ... & Wigneron, J. P. (2024). Anthropogenic disturbance exacerbates resilience loss in the Amazon rainforests. *Global Change Biology*, 30(1), e17006.

42. Liu, T., Morr, A., Bathiany, S., Blaschke, L. L., Qian, Z., Diao, C., ... & Boers, N. (2026). Data gaps and outliers distort critical-slowing-down-based resilience indicators. *Science Advances*, 12(11), eaee1916.

76. Smith, T., & Boers, N. (2023). Reliability of vegetation resilience estimates depends on biomass density. *Nature Ecology & Evolution*, 7(11), 1799-1808.

ED4 - Gap filling clearly biases autocorrelation high. How does that propagate through into your results? You could compare to nan-aware TAC (e.g., np.ma.corrcoeff()) to make sure that isn't a strong factor in your results.

Response: Thank you for this helpful suggestion. In the previous revision, we explicitly assessed the influence of gap filling on the TAC–HSM relationship. Across both bimonthly and quarterly compositing schemes, the strength and direction of the association remained consistent under different gap-filling thresholds, indicating that our main results are robust to interpolation choices.

Reviewer #2 (Remarks on code availability):

The repository is much improved from the previous version, and the Earth Engine codes look fine to me.

Response: Thank you!
